# One-way SMS and healthcare outcomes in Africa: Systematic review of randomised trials with meta-analysis

**DOI:** 10.1371/journal.pone.0217485

**Published:** 2019-06-06

**Authors:** Ditte S. Linde, Malene Korsholm, Johnson Katanga, Vibeke Rasch, Andreas Lundh, Marianne S. Andersen

**Affiliations:** 1 Department of Clinical Research, University of Southern Denmark, Odense, Denmark; 2 Department of Obstetrics and Gynaecology, Odense University Hospital, Odense, Denmark; 3 Odense Patient Data Explorative Network (OPEN), University of Southern Denmark, Odense, Denmark; 4 Danish Centre for Health Economics (DaCHE), Department of Public Health, University of Southern Denmark, Odense, Denmark; 5 Department for Cancer Prevention Services, Ocean Road Cancer Institute, Dar es Salaam, Tanzania; 6 Centre for Evidence-Based Medicine (CEBMO), Odense University Hospital, Odense, Denmark; 7 Department of Infectious Diseases, Hvidovre Hospital, Hvidovre, Denmark; 8 Department of Medical Endocrinology, Odense University Hospital, Odense, Denmark; University of the Witwatersrand, SOUTH AFRICA

## Abstract

**Background:**

The impact of one-way SMS on health outcomes in Africa is unclear. We aimed to conduct a systematic review of one-way SMS randomised trials in Africa and a meta-analysis of their effect on healthcare appointments attendance and medicine adherence.

**Methods:**

PubMed, Embase, CENTRAL, The Global Health Library, ClinicalTrials.gov, ICTRP, and PACTR were searched for published and unpublished trials in Africa without language restriction (up to April 2018). Trials reporting effect estimates on healthcare appointment attendance and medicine adherence were assessed for risk of bias and included in meta-analyses using random-effects models. Other outcomes were reported descriptively. The protocol is registered in PROSPERO, ID:CRD42018081062.

**Results:**

We included 38 one-way SMS trials conducted in Africa within a broad range of clinical conditions. Eighteen trials were included in the meta-analyses, and four were assessed as overall low risk of bias. One-way SMS improved appointment attendance, OR:2·03; 95% CI:1·40–2·95 (12 trials, 6448 participants), but not medicine adherence, RR:1·10; 95% CI:0·98–1·23 (nine trials, 4213 participants). Subgroup analyses showed that one-way SMS had the highest impact on childhood immunization attendance, OR:3·69; 95% CI:1·67–8·13 (three trials, 1943 participants). There was no clear evidence of one-way SMS improving facility delivery, knowledge level (reproductive/antenatal health, hypertension), diabetes- and hypertension management.

**Conclusion:**

In an African setting, the clinical effect of one-way SMS is uncertain except for appointment attendance where the effect seems to vary depending on which clinical condition it is used in.

## Introduction

Mobile health (mHealth) interventions have a growing focus within global health research as these interventions have the potential to reach underserved communities and remote populations in innovative ways [1]. mHealth is defined as the use of mobile and wireless technologies for health [2] and involves different communication channels including one- or two-way Short Message Service (SMS), applications (app)s, and mobile phone calls targeted healthcare clients or -professionals [3]. Moreover, the content and the length of the SMS may vary and can include reminders, education or a combination. This review concerns one-way SMS, which means that the receiver cannot respond to the SMS. It is the simplest form of mHealth as it does not allow for interaction between the sender and receiver, thus it can be implemented in most settings with minimum costs [4].

Few systematic reviews have been published on mHealth interventions in “low- or middle-income countries” (LMIC) [5–7], and the effect of one-way SMS in this setting is unclear. This can be due to the reviews including all forms of mHealth interventions and looking across too diverse populations and settings. To better estimate the effect of one-way SMS, it may be relevant to make a regional restriction, apart from an economic restriction, as digital literacy, network infrastructure, and cultural/social acceptance of mHealth interventions may be more homogenic within a certain region [3]. As all countries in Africa have moderately comparable economies—all countries are “LMIC” apart from the Seychelles [8]–it is relevant to look at this continent specifically. A series of Cochrane reviews published between 2012–2017 assessed the effect of SMS on various health issues with no restriction on type of setting [9–14]. Only two of these reviews included trials from Africa and concluded that SMS was effective in improving healthcare appointment attendance and HIV medicine adherence [12,13]. However, of the 10 trials included in these two reviews, only two trials concerned one-way SMS interventions in Africa, both with evidence [12,13] of one-way SMS improving healthcare appointment attendance [15] and HIV medicine adherence [16].

As there remains a lack of evidence of the effect of one-way SMS in resource-limited settings, we conducted a systematic review of one-way SMS trials in Africa and a meta-analysis of their effect on healthcare appointment attendance and medicine adherence. This review will provide an overview of the effect of one-way SMS among different clinical conditions in Africa and may help clarify which health areas this separate element of mHealth should be prioritised in future mHealth strategies and policies in Africa.

## Material and methods

For this systematic review and meta-analysis, we searched PubMed, Embase, Cochrane Central Register of Controlled Trials (CENTRAL), and The Global Health Library for trials published in any language (from inception up to 18 April 2018; S1 File). The search strategy was developed in collaboration with an information specialist and included search terms such as “trial” AND “Africa” AND “text message” OR “sms” OR “mobile phone intervention”. We searched relevant reviews and reference lists of included trials and ClinicalTrials.gov (April 2018), the International Clinical Trial Registry Platform (ICTRP) (April 2018), and the Pan African Clinical Trial Registry (PACTR) (Oct 2018) for additional eligible and ongoing or unpublished trials. United Nations and World Bank databases were searched for reports containing relevant trials (May 2018). Our protocol was registered in PROSPERO prior to study conduct (ID: CRD42018081062, 10 January 2018).

After removing duplicates, two authors independently screened titles and abstracts (DSL, JK) and full-text (DSL, MK) using Covidence (www.covidence.org/). Disagreements were resolved through discussion. We included published and unpublished randomised controlled trials (RCTs) in any language, including cluster- and pilot RCTs. The setting was limited to Africa, and trial participants could be all types of healthcare clients including guardians for healthcare clients. We included interventions that used SMS to affect healthcare behaviour and health knowledge. At least one intervention arm had to be exclusive one-way SMS, which the participant could not respond to. Included trials had to have a control group that received standard care, no- or placebo SMS. If co-interventions (e.g. written material) were received by participants in both intervention and control arms, this was considered to be part of standard care, and such trials were included.

One author (DSL) extracted data into a standardised Excel template and one co-author (MK) verified outcome data. Extracted data included: title, first author, publication year, journal/register, randomisation method, clinical conditions, setting, country, number of participants, gender distribution, inclusion/exclusion criteria, description of intervention/controls, study period, outcomes measures, and outcomes for finished trials. Twenty-six corresponding authors were contacted for clarification or to obtain missing data.

### Risk of bias assessment

For trials that assessed the effect of SMS on healthcare appointment attendance or medicine adherence, two authors (DSL, MK) independently assessed risk of bias using the Cochrane Risk of Bias Tool [17]. We assessed the domains: random sequence generation and allocation concealment (selection bias), blinding of personnel (performance bias), blinding of outcome assessment (detection bias), incomplete reporting (attrition bias) and selective reporting (reporting bias). The domains were judged to have either low risk, high risk, or unclear risk of bias. In case of disagreement, another co-author (AL) was used as arbiter. Due to the overt nature of the intervention, study participants were not blinded. Therefore, judgement of performance bias was based solely on blinding of personnel. We did not assess risk of attrition bias on trials that only had healthcare appointment attendance as an outcome as incomplete outcome data was part of this outcome, i.e. non-attendance resulted in loss to follow-up (i.e. resulted in blank cells in risk of bias assessment). Cluster trials were additionally judged for risk of baseline imbalance and recruitment bias [17]. Trials were judged to have overall low risk of bias, if they scored low risk in selection, detection, and reporting bias. All other trials were considered to be overall high-risk of bias trials.

### Data analysis

We expected that one-way SMS interventions were used in various types of populations and settings and therefore including multiple types of outcomes. Accordingly, we performed an overall descriptive analysis of all trials and performed meta-analyses restricted on the outcomes “appointment attendance” and “medicine adherence”, which we regarded to be uniform. For our descriptive analysis, we reported unadjusted trial results on the primary outcome. If no quantitative estimates were reported for dichotomous data we calculated risk ratios (RRs), if possible.

Meta-analyses were done using Reviewer Manager 5·3 [18]. Due to anticipated clinical and methodological heterogeneity, we planned to use a random-effects model with the Mantel-Haenszel method for dichotomous data to calculate pooled RRs and estimate 95% confidence intervals (CIs) for both appointment attendance and medicine adherence. However, one trial^19^ assessing appointment attendance was randomised at cluster level. The trial results were reported in odds ratios (OR) and analysed taking clustering into account, thereby avoiding unit-of-analysis error. For appointment attendance, we therefore calculated a pooled estimate in OR using the generic-inverse variance method to allow for the inclusion of this trial. The pooled estimate for medicine adherence was calculated as RR as planned. Heterogeneity was assessed using I^2^.

We performed subgroup analyses comparing overall low risk of bias trials with high risk of trials, and clinical conditions. We performed sensitivity analyses using fixed-effect models and excluding the cluster randomised trial [19]. Additionally, in our analysis on appointment attendance, one trial was an outlier with an extreme result [20]. We discovered that this trial was published in a journal on Beall’s list of potential predatory publishers [21]. We then assessed all trials and discovered another of the included trials being published in a journal on Beall’s list [22]. We then did post-hoc sensitivity analyses excluding both trials.

## Results

**Fig 1:**
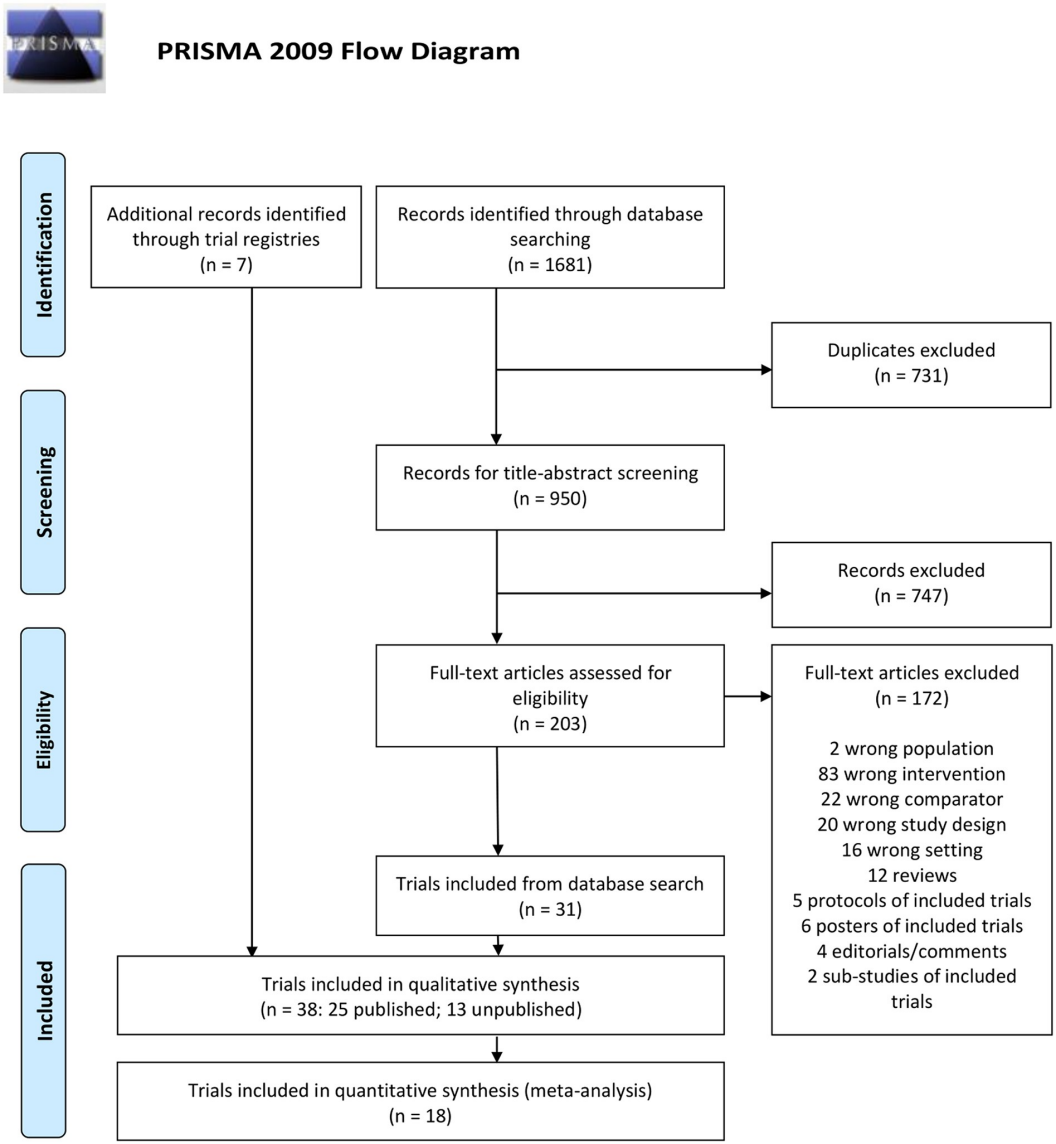
PRIMSA flow diagram. *From*: Moher D, Liberati A, Tetzlaff J, Altman DG, The PRISMA Group (2009). *P*referred *R*eporting *I*tems for *S*ystematic Reviews and *M*eta-*A*nalyses: The PRISMA Statement. PLoS Med 6(7): e1000097. doi:10.1371/journal.pmed.1000097. **For more information**, **visit** www.prisma-statement.org.

We identified 1681 records in our database search. After excluding 731 duplicates, 747 records were excluded following title-abstract screening (Fig 1). A total of 203 records were reviewed in full-text and 172 were excluded. This led to an inclusion of 31 trials [15,16,19,22–49]. Searching other sources led to the inclusion of seven additional trials [20,50–55]. In total, we included 38 trials, of which 25 were published [15,16,19,20,22–42], and of the 13 unpublished trials, nine were ongoing [46–52, 54, 55), three were finished [43,44,53], and one was interrupted before enrolment of all participants [45].

**Fig 2:**
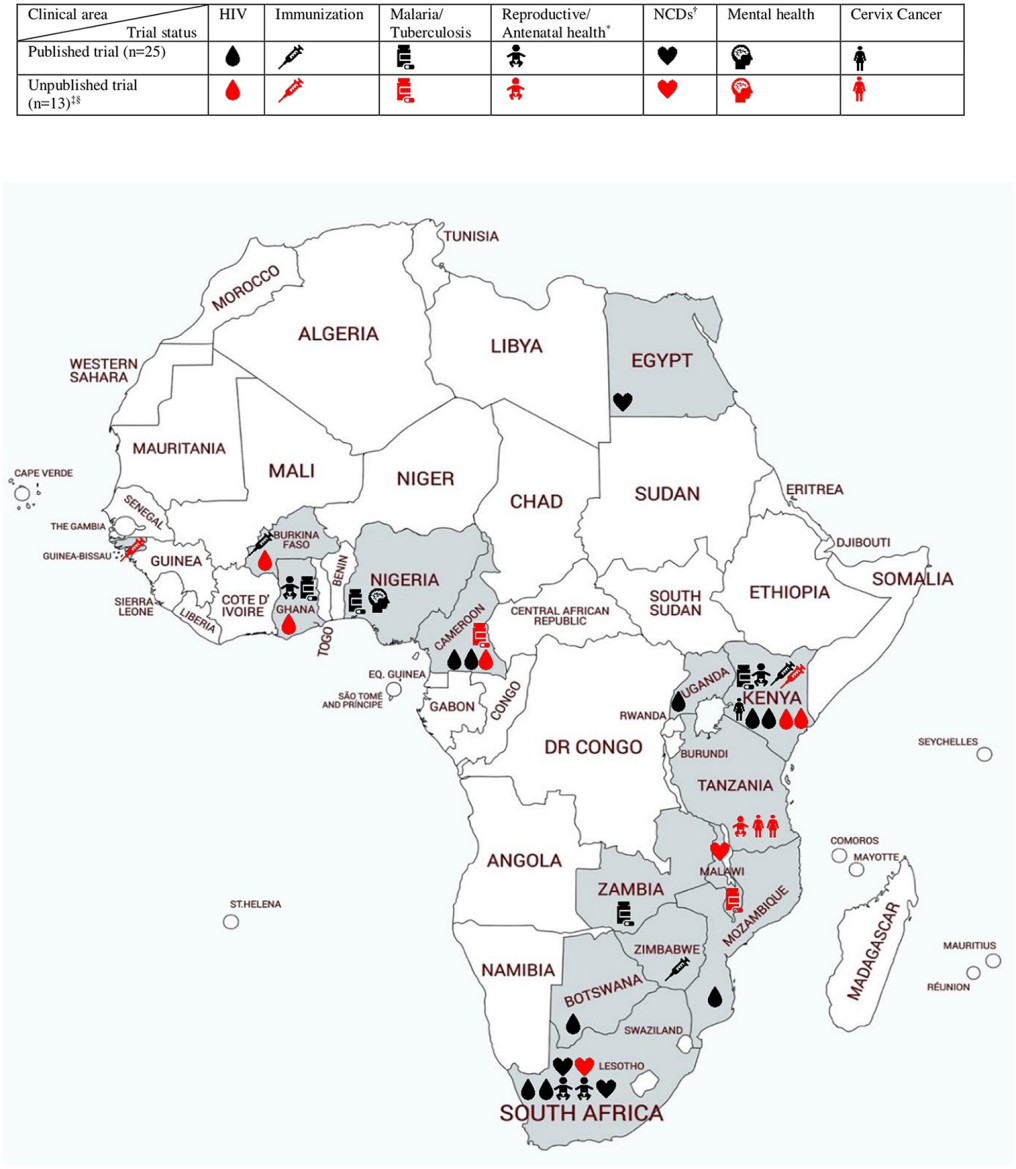
One-way SMS trials in Africa: Trial status and clinical conditions. *Reproductive/antenatal health: Reproductive/antenatal knowledge, medical abortion, facility delivery, child dietary diversity. ^†^Non-communicable diseases: Diabetes, hypertension. ^‡^One ongoing NCD trial is a multicenter study in South Africa and Malawi, hence the number of symbols exceeds the number of included trials by one. ^§^Map developed by use of mapchart.net.

Trials were published between 2011–2018, and a total of 15438 participants were included (median: 304 participants) [15,16,19,20,22–42] (Table 1). In the unpublished and ongoing trials (excluding an interrupted and cluster trial), a total of 10783 participants were planned to be enrolled (median: 600 participants) [43,46–55]. Age among participants ranged from 45 days to 54 years. Five trials targeted infants and children where the caregiver was the receiver of the SMS intervention. All, but one trial, were set in Sub-Saharan Africa; 25 in East and Southern Africa, 12 in Western Africa, and one Northern Africa (Fig 2). Sixteen out of 38 trials were set in either Kenya or South Africa. Settings ranged from rural outpatient clinics and health centres to regional hospitals and drug shops.

**Table 1:** One-way SMS trials in Africa.

	Country	Clinical area	Trial size (n)	Female	Age (Mean)	Follow-up (weeks)	Primary endpoint*	Intervention A (type)	Effect of intervention on primary endpoint compared to control
								Intervention B	
Finished trials, published									
2018 (Unger) [33]	Kenya	Antenatal care	298	100%	23 *median*	24	Facility delivery	SMS^†^ (E^§§^+M^§§^)	RR = 1.0 [95% CI: 1.0 to 1.0]
								SMS+quiz	RR = 1.0 [95% CI: 1.0 to 1.0]
2017 (Abaza) [23]	Egypt	Diabetes	73	56%	51.5	12	ΔHbA1c	SMS (E+R^§§^)	Δ0.29 [95% CI: -0.4 to 1.0]
2017 (Linnemayr) [24]^§^	Uganda	HIV	332	60%	18.3	52	Medicine adherence	SMS (M)	Proportion taken/total prescribed = 0.64, (p = 0.27)
								Two-way SMS	Proportion taken/total prescribed = 0.61, (p = 0.15)
2017 (Reid) [25]^§^	Botswana	HIV	108	44%	41.1	47	Medicine adherence	SMS (R)	OR = 2.4 [95% CI:0.9 to 6.4]
2017 (Rokicki) [26]	Ghana	Reproductive knowledge	756	100%	17.7	12	Increase knowledge *(Pregnancy prevention/ STD*^‡‡^*)*	SMS (E)	11% higher than control [95% CI: 7 to 15%]
								SMS+quiz	24% higher than control [95% CI: 19 to 28%] *24-item true/false question*
2017 (Talisuna) [27]^§^	Kenya	Malaria *(infant)* ^¶^	1677	47%	*categories*^‡^	4	Medicine adherence	SMS (R)	OR = 1.1 [95% CI: 0.4 to 3.3]
2017 (Thomas) [28]^§^	Nigeria	Psychosis	200	54%	33.7	2–4	Attendance follow-up appointment	SMS (R)	OR = 1.8 [95% CI:1.0 to 3.2]
2017 (Wanyoro) [20]^§^	Kenya	Cervical cancer screening	286	100%	38.8	52	Attendance follow-up screening	SMS (R)	OR = 8.0 [95% CI:4.7 to 13.7]
2016 (Bobrow) [29]^§^	South Africa	Hypertension	1372	72%	54.3	52	Δsystolic blood pressure	SMS (E+M+R)	-2.2mmHg [95% CI: -4.4 to -0.04]
								SMS+two-way SMS	-1.6mmHg [95% CI: -3.7 to 0.6]
2016 (Davey) [30]^§^	Mozambique	HIV	830	60%	36.9 *median*	52	Appointment attendance	SMS (E+R)	RR = 1.0 [95%CI: 1.0 to 1.1]**
2016 (Hacking) [31]	South Africa	Hypertension knowledge	223	80%^¶^	52.8	17	Increase knowledge *(Hypertension)*	SMS (E+R)	Score = 17.5, (p = 0.69) *19 questionnaire items*, *max score = 19*
2016 (Haji) [19]^§^	Kenya	Childhood immunization^¶^	1116	49%	45 days *median*	16	Vaccination attendance *(3*^*rd*^ *dose)*	SMS (R)	OR = 5.6 [95% CI: 3.0 to 10.4]
								Sticker	OR = 1.1 [95% CI: 0.7 to 1.6]
2016 (Liu) [32]^§^	Nigeria	Malaria	686	42%	32.8	4 days	Medicine adherence	Short SMS (E+T^§§^)	OR = 1.4 [95% CI: 0.9 to 2.2]
								Long SMS (E+E+T)	OR = 1.1 [95% CI: 0.7 to 1.5]
2016 (Nsagha) [22]^§^	Cameroon	HIV	90	61%	38.8	4	Medicine adherence	SMS (E)	RR = 1.5 [95% CI: 1.0 to 2.2]**
2016 (Steury) [34]^§^	Zambia	Malaria	96	48%	*categories*^‡^	1	Medicine adherence	SMS (R)	RR = 0.9 [95% CI: 0.7 to 1.3]**
2015 (Bangure) [35]^§^	Zimbabwe	Childhood immunization^¶^	304	100% *mothers*	26.5 *median*	14	Vaccination attendance *(3*^*rd*^ *dose)*	SMS (E+R)	RR = 1.3 [95% CI: 1.1 to 1.4]**
2015 (Orrell) [36]^§^	South Africa	HIV	230	65%	34.5	48	Medicine adherence	SMS (R)	aOR = 1.1 [95% CI:0.8 to 1.5]
2015 (Sclumberger) [37]^§^	Burkina Faso	Childhood immunization^¶^	523	100% *mothers*	*unknown*	52	Vaccination attendance *(3*^*rd*^ *dose)*	SMS (R)	RR = 1.4 [95% CI: 1.9 to 1.6]**
2014 (Bigna) [38]^§^	Cameroon	HIV *(infant)*^¶^	242	85%	42.8	*unknown*	Attendance follow-up appointment	SMS (R)	OR = 2.9 [95% CI:1.3 to 6.3]
								Call	OR = 5.5 [95% CI:2.3 to 13.1]
								SMS+call	OR = 7.5 [95% CI:2.9 to 19.0]
2014 (Constant) [39]	South Africa	Medical abortion	469	100%	25.8	12	Decrease anxiety level	SMS (E+R)	Absolute difference = 1.3, p = 0.01 *HADScale with 14 items each scored 0–3*
2014 (Lau) [40]	South Africa	Antenatal knowledge	206	100%	27.0	40	Increase in knowledge	SMS (E+R)	Mean = 10.2 [95% CI:9.8 to 10.6] *9 questionnaire items*, *max score*: *18*
2014 (Raifman) [41]^§^	Ghana	Malaria	1140	55%^¶^	*categories*^‡^	3 days	Medicine adherence	SMS A (R)	aOR = 1.5 [95% CI: 1.0 to 2.0] OR = 1.24 [95% CI: 1.0 to 1.6]^††^
								SMS A+B (M+R)	aOR = 0.8 [95% CI: 0.5 to 1.2] OR = 1.1 [95% CI: 0.8 to 1.5]^††^
2012 (Odeny) [15]^§^	Kenya	HIV prevention	1200	0%	24.9 *median*	7 days	Attendance post-circumcision appointment	SMS (E+R)	RR = 1.1 [95% CI: 1.0 to 1.2]
2012 (de Tolly) [39]	South Africa	HIV	2553	*unknown*	*unknown*	3	HIV testing	3xSMS (E)	OR = 0.9 [95% CI: 0.7 to 1.3]
								10xSMS (E)	OR = 1.1 [95% CI: 0.8 to 1.4]
								3xSMS (M)	OR = 0.7 [95% CI: 0.5 to 1.0]
								10xSMS (M)	OR = 1.7 [95% CI: 1.2 to 2.4]
2011 (Pop-Eleches) [16]^§^	Kenya	HIV	428	66%	36.3	48	Medicine adherence	Short daily SMS (R)	RR = 1.0 [95% CI:0.7 to 1.4]**
								Long daily SMS (M+R)	RR = 1.0 [95% CI: 0.6 to 1.9]**
								Short weekly SMS	RR = 1.3 [95%:1.0 to 1.8]**
								Long weekly SMS	RR = 1.3 [95% CI:1.0 to 1.8]**
Finished trials, unpublished									
2016 (NCT02680613) [53]	Tanzania	Cervical cancer screening	600	100%	-	15	Screening attendance	SMS (M)	-
								SMS+travel voucher	-
2016 (Gibson) [43]	Kenya	Childhood immunization^¶^	2432	100% *mothers*	-	52	Vaccination attendance	SMS (R+M)	-
								SMS+75 shilling	-
								SMS+200 shilling	-
2016 (Rossing) [45] *Interrupted*	Guinea-Bissau	Measles vaccination^¶^	990	100% *mothers*	-	<72	Measles vaccine coverage	SMS (R)	-
								SMS+call	-
2016 (Wagner) [44]	Burkina Faso	HIV	72 *centres*	*unknown*	-	104	Medicine adherence	SMS 1 (R)	-
								SMS 2 (R)	-
								SMS 3 (R) +MMS	-
								MMS	-
Ongoing trials, unpublished									
2018 (PACTR201802003035922) [54]	Cameroon	HIV/ Tuberculosis	228	*unknown*	-	26	Medicine Adherence	1xSMS weekly (R+M)	-
								2xSMS weekly	-
2017 (Drake) [46]	Kenya	HIV	825	100%	-	104	Maternal virologic failure *(RNA>1000)*	SMS (E+M+R)	-
								SMS+quiz	-
2017 (Linde) [47]	Tanzania	Cervical cancer screening	700	100%	-	60	Attendance follow-up screening	SMS (E+R)	-
2017 (NCT03297190) [52]	Tanzania	Diet *(infant)*	2400^||^	100%	-	*unknown*	Consumption of >4 food groups	SMS (E)	-
								Counsel	-
								SMS+counsel	-
2016 (NCT02721420) [51]	Malawi	Anaemia *(child)*^¶^	375	*unknown*	-	15	Medicine adherence	SMS 1 (R)	-
								SMS 2 (R)	-
								Health worker reminder	-
2016 (NCT02915367)[50]	Kenya	HIV	350	100%	-	<104	Medicine adherence	SMS (R)	-
2015(ISRCTN-70768808) [55]	South Africa/ Malawi	Diabetes	1065	*unknown*	-	52	ΔHbA1c	SMS (E+M+R)	-
2015 (L’Engle) [48] *Unknown status*	Ghana	HIV	1600	*unknown*	-	52	Medicine adherence	SMS (R)	-
2014 (Bediang) [49]	Cameroon	Tuberculosis	208	*unknown*	-	32	Cure	SMS (R+M)	-

*Primary endpoint as reported in trial. If several primary endpoints were reported, then the first mentioned is reported in this table.

^†^SMS = One-way SMS unless specified otherwise.

^‡^Talisuna 2017: <1yr = 10%, 1-5yrs = 89%, 5yrs = 1%.

Steury 2016: 18-25yrs = 35%; 26-35yrs = 28%; 36-50yrs = 24%; <50yrs = 16%.

Raifman 2014: SMS/control = 17%/14% (<5yrs), 21%/16% (5-17yrs), 56%/63% (18-59yrs).

^§^Trial eligible for meta-analysis and assessed for risk of bias.

^¶^SMSs sent to mother’s/caregiver’s phone.

^||^Unpublished information received by corresponding author.

**Relative Risk (RR) calculated based on numbers stated in article.

^††^The trial only reports an adjusted OR. An unadjusted OR has been calculated based on numbers stated in article. The second SMS-arm (Long SMS) is a pseudo-randomised intervention arm.

^‡‡^STD: Sexual transmitted disease

^§§^E = Educative SMS; M = Motivational SMS; R = Reminder SMS; T = Test result SMS

The function of the one-way SMS interventions varied from educational and motivational messages to reminders and test results, or a combination of these. For example, a combined educative and reminder message read, “*Immunization protects your child against killer diseases such as polio*, *whooping cough*, *diphtheria*, *measles*, *pneumonia and tuberculosis*. *You are reminded that the vaccination appointment will be due in 7 days from today”* [35] while a motivational message could be, “*If you test and you’re HIV+ you can go on free drugs when you need to*. *HIV is longer a death sentence*. *You can live a long*, *normal life with HIV*. *Plz test*!*”* [39]. Most trials only sent SMS reminders [16,19,20,24,25,27,28,36–38,41,44,45,48,50,51] or reminders combined with educative messages [15,23,30,31,35,39,40,47] (Table 1). Two trials reported that the SMS content was developed based on behavioural theories [39,53] and 16 trials reported that the SMS content had been pre-tested or developed in consultation with experts, clinical staff and/or potential participants [16,24–27,29–33,36,40,41,46–48]. Clinical conditions included HIV (n = 13), immunization (n = 5), reproductive and antenatal health (n = 5), and malaria (n = 4). Fourteen trials [16,22,24,25,27,32,34,36,41,44,48,50,51,54] had medicine adherence as primary outcome though it was measured in various ways including timely pick-up of medicine, pill counts, self-reported behaviour, and pillbox openings. Eleven trials [15,19,20,28,30,35,37,38,43,47,53] had appointment attendance as primary outcome, which included attendance to childhood vaccinations, screening, medical follow-up appointments and proxy measures for retention in HIV care. Four trials had surrogate outcomes as primary outcome (i.e. ΔHbA1c, Δblood pressure, RNA>1000) [23,29,46,55], three trials had knowledge change as primary outcome [26,31,40], and six trials had either facility delivery[33], HIV test [42], tuberculosis cure [49], measles vaccine coverage [45], decrease in anxiety level [39], or consumption of >4 food groups [52] as primary outcome.

Seven published trials had outcomes that did not include adherence or attendance [23,26,31,33,39,40,42] and their primary outcome was therefore only analysed descriptively. One trial had a surrogate primary outcome (Δblood pressure) and secondary outcomes on adherence and attendance [29] and included in both the descriptive- and meta-analysis. One [26] of three trials on knowledge change found an intervention effect as one-way SMS increased reproductive health knowledge among adolescent girls with 11% (95% CI: 7–15%) compared to controls (3 months after baseline). Yet, knowledge increased with 24% (95% CI: 19–28%) if they also received an interactive SMS quiz. However, at 15 months follow-up, there was no difference in knowledge level (3%; 95% CI:-1% to 7%) between the one-way SMS group and controls. One trial [39] found one-way SMS decreased anxiety after medical abortion when measured on a HADScale (absolute difference 1·3, p = 0·01). Additionally, one trial [42] found that one type of one-way SMS (10 motivational SMS) increased HIV testing (OR = 1·7, 95% CI: 1·2–2·4) while the other three types of one-way SMS had no effect compared to controls. Trials on facility delivery [33], diabetes management [23], and hypertension [29,31] found no statistically significant effect of one-way SMS on the primary outcomes.

### Meta-analysis and risk of bias assessment

**Fig 3:**
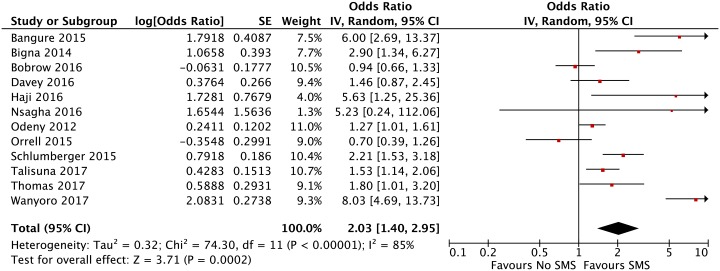
Effect of one-way SMS versus no SMS on healthcare appointment attendance.

**Fig 4:**
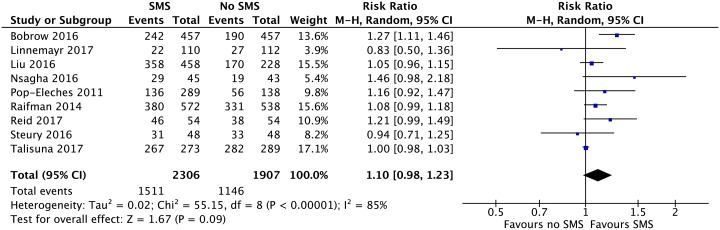
Effect of one-way SMS versus no SMS on medicine adherence.

Eighteen published trials all set in Sub-Saharan Africa could be included in the meta-analysis and judged for risk of bias. Twelve trials [15,19,22,20,27–30,35–38] (6448 participants) were included in our pooled analysis on healthcare appointment attendance. We found that one-way SMS improved appointment attendance compared with no SMS, OR: 2·03; 95% CI: 1·40–2·95; I^2^ = 85% (Fig 3). Nine trials [16,22,24,25,27,29,32,34,41] (4213 participants) were included in our pooled analysis on medicine adherence. Data from one additional trial could not be included as adherence was measured as a continuous outcome [36]. We found that one-way SMS did not improve medicine adherence compared with no SMS, RR: 1·10; 95%: 0·98–1·23; I^2^ = 85% (Fig 4). For appointment attendance, sensitivity analysis showed a somewhat lower treatment effect using a fixed effect model compared to a random effects model, Fixed OR: 1·62; 95% CI: 1·42–1·85 versus Random OR: 2·03; 95% 1·40–2·95 (Fig A in S2 File). For medicine adherence the effect estimates were similar, however, they became statistically significant using the fixed effect model, Fixed RR: 1·09; 95% CI: 1·05–1·14 versus Random: RR 1·10; 95%: 0·98–1·23 (Fig F in S3 File). A sensitivity analysis excluding the cluster trial [19] gave similar results as our primary analysis on appointment attendance. Post hoc sensitivity analyses excluding the trials [20,22] from potentially predatory journals did not alter our previous findings. We still found a statistically significant effect of SMS on appointment attendance, though the effect estimate and heterogeneity decreased, OR: 1·66; 95% CI: 1·23–2·24, I^2^ = 76% (Fig C in S2 File), and no statistically significant effect of one-way SMS on medicine adherence, RR: 1.08; 95% CI: 0·97–1·21, I^2^ = 85% (Fig I in S3 File).

**Fig 5:**
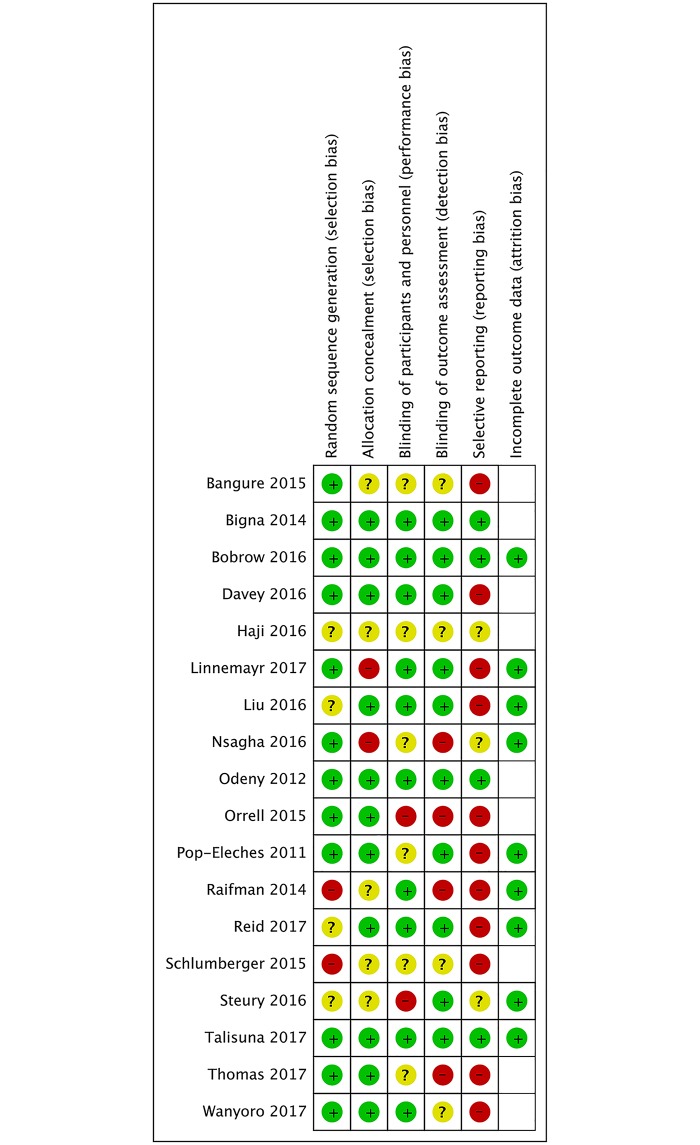
Risk of bias assessment*. *Empty cell: No bias assessment.

Four trials [15,27,29,38] were judged as overall low risk of bias trials and 14 as high risk of bias trials [16,19,20,22,24,25,28,30,32,34–37,41] (Fig 5; S4 File).When comparing low risk of bias trials with high risk of bias trials, we found a lower treatment effect on appointment attendance in low risk of bias trials, OR: 1·36; 95% CI: 1·01–1·84; I^2^ = 66% versus OR: 2·62; 95% CI: 1·42–4·83; I^2^ = 85% (interaction test, p = 0·06) (Fig D in S2 File). When comparing low risk of bias trials with high risk of trials, we found no difference in effect on medicine adherence, RR: 1·13; 95% CI: 0·63–2·03; I^2^ = 99% versus RR: 1·08; 95% CI: 1·02–1·14; I^2^ = 2% (interaction test, p = 0.09). However, when stratifying the analysis in relation to risk of bias, heterogeneity disappeared in the high risk of bias group and increased in the low risk of bias group (Fig G in S3 File).

When stratifying data on appointment attendance according to clinical conditions, the analysis showed that SMS had an effect on childhood immunization (n = 3 trials) but not on HIV appointment attendance (n = 4 trials), OR: 3·69; 95% CI: 1·67–8·13 versus OR: 1·48; 95% CI: 0·73–3·00. The remaining trials had different clinical conditions and showed differential effects (Fig E in S2 File). When stratifying data on medicine adherence according to clinical conditions, the analysis showed that SMS had an effect on HIV medicine adherence (n = 4 trials) but not on malaria medicine adherence (n = 4 trials), RR: 1·18; 95% CI: 1·02–1·37 versus RR: 1·04; 95% CI: 0·94–1·12. The last trial concerned adherence to hypertension medicine and found an effect, RR: 1·27; 95% CI: 1·11–1·46 (Fig H in S3 File).

## Discussion

In this systematic review and meta-analysis of one-way SMS trials in Africa, we found that one-way SMS overall improved healthcare appointment attendance though not medicine adherence. When stratifying data according to clinical conditions, our results suggest that one-way SMS has the highest impact on attendance to childhood immunization appointments. Additionally, our subgroup analysis suggests a minor impact on HIV medicine adherence and no impact on malaria medicine adherence. The majority of trials were assessed as high risk of bias and there was a non-statistically significant trend of lower effect estimates in the group of low risk of bias trials suggesting that the true effect of one-way SMS on appointment attendance may be lower than what we found in our primary analysis. Our descriptive analysis found no clear evidence of one-way SMS improving facility delivery, knowledge levels (reproductive/antenatal health, hypertension) or diabetes- and hypertension management. One-way SMS has been used in 38 trials across Africa. All trials were set in Sub-Saharan Africa except one from Egypt, and the majority of trials were set in South Africa and Kenya.

To our knowledge, this is the first review that provides an overview of the effect of one-way SMS on various health outcomes in Africa. In contrast to other reviews on mHealth interventions, we have chosen only to limit our review in regard to type of intervention (one-way SMS) and setting (Africa), whilst other systematic reviews tend to focus on how various types of SMS interventions overall affects a specific clinical condition across a wider clinical setting. We chose this set of limits as adaption to mHealth interventions may be affected by local conditions [3], hence it is plausible that they have differential effect across settings. Further, the inclusive approach among other reviews on mHealth interventions entail that the effect of specific elements of mHealth, such as one-way SMS, is unclear. Our choice has limited our ability for in-depth analysis of specific clinical conditions but allowed us to provide a nuanced overview of how one-way SMS works generally in Africa. This can guide future mHealth research and strategies within Africa.

Our meta-analysis indicates that one-way SMS appears to have differential effect across clinical conditions, which is not surprising. It is likely more acceptable and manageable to attend short-term childhood vaccination appointments than life-long HIV appointments. HIV-related stigma is still an issue in Africa [56] and a basic one-way SMS may not able to overcome HIV-related barriers. Different types of mHealth interventions that include counselling or two-way SMS, where the receiver can communicate with the healthcare provider, may be more effective. However, it is outside the scope of this review to assess this type of mHealth interventions. Furthermore, a limitation of our subgroup analysis on clinical conditions is that outcomes were measured heterogeneously across trials. Despite our analysis indicating that one-way SMS has an effect on “HIV medicine adherence”, adherence was measured in various ways such as pick-up of medicine, pill counts, self-reported behaviour and automated pill boxes. Hence, these various differential outcome measures may be too diverse to group into one category. Though our analysis showed minor heterogeneity with an I^2^ = 7% (Fig H in S3 File).

In our descriptive assessment of one-way SMS, we have stringently used unadjusted effect estimates and a 5% significance level (Table 1), which at times resulted in different conclusions than what was concluded by the trial authors [16,41]. E.g., a trial from Kenya concluded that one-way SMS improved HIV medicine adherence based on pooling two intervention arms (short/long weekly SMS) compared to controls (53% versus 40% , p = 0·03) [16]. However, individually these two intervention arms were not significantly different from the control group, RR: 1·3, 95% CI:1·0–1·8, p = 0·07 (short weekly SMS) and RR: 1·3, 95% CI: 1·0–1·8, p = 0·08 (long weekly SMS). Further, if all four intervention arms are pooled (short/long daily/weekly SMS) the effect is not significant either, RR: 1·2, 95% CI: 0·9–1·5. Our analytical strategy is more conservative as some of the strategies employed by trial authors did not seem to be pre-specified. This highlights the importance of transparently reporting the choice of analytical strategy as this affects the overall conclusions of the effectiveness of one-way SMS. Additionally, our assessments showed that few trials were guided by health behaviour theory. Health behaviour theories, such as “The health belief model”, “The theory of planned behaviour”, and “The transtheoretical model and stages of change”, may increase the likelihood of interventions succeeding as they can help understand *why* people behave as they do, *what* researchers need to know before developing an intervention, and *how* interventions can be shaped so that they impact the target group as much as possible [57]. It is plausible that one-way SMS interventions may be more effective if researchers have a theoretical approach to developing the interventions.

The comparability of our findings is limited as other systematic reviews have had more inclusive approaches to mHealth and SMS. A 2013 Cochrane Review on SMS reminders and attendance to healthcare appointments concluded that there was low to moderate quality evidence of reminders increasing attendance compared to no or postal reminders, RR: 1·14, 95% CI: 1.03–1.26 [12]. This finding is in line with our results despite the evidence mainly stems from different settings; only one African trial was included in the Cochrane Review [15]. This was partly due to the Cohrane review excluding trials where the reminder was sent to the caretaker—e.g. in the case of childhood immunization—and partly due to to most African one-way SMS trials being published after the Cochrane Review. A 2015 systematic review on mHealth interventions’ effect on antenatal, postnatal and childhood immunization in LMIC^6^ included both SMS and apps targeted pregnant women or healthcare workers. No trials on childhood immunization were included though three observational studies—from Kenya [58,59] and Malawi [60]—found that SMS increased immunization rates. However, the quality of evidence was low to moderate. These findings are also in line with the results of our review.

A 2012 Cochrane Review on mobile messaging and HIV medicine adherence included two trials from Kenya and concluded there was high quality evidence of SMS enhancing adherence to anti-retroviral therapy, RR: 1.16, 95% CI: 1.02–1.32 [13]. As the point estimate is similar to ours, this result supports our finding that one-way SMS have modest effect on HIV adherence, yet we did not find high-quality evidence as all four trials on HIV medicine adherence were assessed as overall high risk of bias. A 2018 systematic review on the effect of voice calls and SMS on HIV medicine adherence also concluded that SMS improved adherence to HIV medicine compared with controls, yet the effect estimate is somewhat higher than what we found, OR: 1·59, 95% CI: 1·3–2·0 [61]. This may be due to the SMS trials included in that review mainly involve two-way SMS or one-way SMS combined with co-interventions. Hence, these may be more effective at improving HIV medicine adherence than one-way SMS. No systematic reviews were found on mHealth and adherence to malaria medication.

From an overall global health perspective, it may be argued that future mHealth strategies and policies in Africa should prioritise to establish one-way SMS within areas, such as childhood immunization programs, as it appears more effective than on medicine adherence. However, as most trials had high risk of bias, there is a need for more large-scale high-quality trials in Africa. As mHealth is a heterogenous field and so is the sub-element of SMS, we recommend that scholars’ approach mHealth and SMS more narrowly and clearly distinguish between different interventions in order to provide a clearer overview of what have proven to work in what contexts within what health outcomes.

## Conclusions

Despite the intriguing nature of simple one-way SMS and their potential to address global health issues in innovative ways, this review found that there is only evidence for the effect of one-way SMS within some outcomes and clinical conditions in Africa. Overall, one-way SMS improves attendance to healthcare appointments but not medicine adherence, and it has highest impact on attendance to childhood immunization appointments. One-way SMS may have modest impact on HIV medicine adherence and we found no evidence of one-way SMS impacting malaria medicine adherence or HIV appointment attendance. We recommend future mHealth strategies and policies in Africa to prioritise to use one-way SMS within childhood immunization programs and reconsider using it on medicine adherence as there is very minor or no effect within this area. However, more high-quality trials are needed. To clearly understand what type of mHealth works in different contexts, we advocate that scholars start differentiating between different types of mHealth and SMS interventions as well as have a theoretical approach when developing the content of the intervention.

## Supporting information

S1 File Literature search strings. (DOCX)

S2 File Subgroup and sensitivity analyses of one-way SMS versus no SMS on healthcare appointment attendance. (DOCX)

S3 File Subgroup and sensitivity analyses of one-way SMS versus no SMS on medicine adherence. (DOCX)

S4 File Risk of bias assessment. (DOCX)

S5 File Protocol. (PDF)

S6 File PRISMA checklist. (DOC)

## Abbreviations

App: Application
CI: Confidence interval
CENTRAL: Cochrane Controlled Register of Trials
ICTRP: International Clinical Trial Registry Platform
LMIC: Low- and middle-income countries
MMS: Multi-media message service
NCD: Non-communicable disease
OR: Odds Ratio
PACTR: Pan African Clinical Trial Registry
RCT: Randomised controlled trial
RR: Risk ratio
SMS: Short message service
