# Supplementary material for: One-way SMS and healthcare outcomes in Africa: Systematic review of randomised trials with meta-analysis
Source: PLoS One. 2019 Jun 6;14(6):e0217485. doi: 10.1371/journal.pone.0217485 (PMC6553734; doi:10.1371/journal.pone.0217485)
Supplement: S1 File — (DOCX) [file pone.0217485.s001.docx]

**S1 File: Literature search strings**

**PUBMED**

(((((((((((((((((((((((((((((((((((((((((((((((((((((((((((("Africa South of the Sahara") OR "Africa, Eastern") OR "Africa, Northern") OR "Africa, Southern") OR "Africa, Western") OR "Africa") OR "African") OR "Algeria") OR "Angola") OR "Benin") OR "Botswana") OR "Burkina Faso") OR "Burundi") OR "Cameroon") OR "Cape Verde") OR "Central African Republic") OR "Chad") OR "Congo") OR "Cote d'Ivoire") OR "Democratic Republic of the Congo") OR "Djibouti") OR "Egypt") OR "Equatorial Guinea") OR "Eritrea") OR "Ethiopia") OR "Gabon") OR "Gambia") OR "Ghana") OR "Guinea-Bissau") OR "Guinea") OR "Kenya") OR "Lesotho") OR "Liberia") OR "Libya") OR "Malawi") OR "Mali") OR "Mauritania") OR "Morocco") OR "Mozambique") OR "Namibia") OR "Niger") OR "Nigeria") OR "Rwanda") OR "Senegal") OR "Sierra Leone") OR "Somalia") OR "South Africa") OR "South Sudan") OR "Sudan") OR "Swaziland") OR "Tanzania") OR "Togo") OR "Tunisia") OR "Uganda") OR "Zambia") OR "Zimbabwe")) OR (((((((((((((((((((((((((((((((((((((((((((((((((((((((("Africa South of the Sahara"[Mesh]) OR "Africa, Central"[Mesh]) OR "Africa, Eastern"[Mesh]) OR "Africa, Northern"[Mesh]) OR "Africa, Southern"[Mesh]) OR "Africa, Western"[Mesh]) OR "Africa"[Mesh]) OR "Algeria"[Mesh]) OR "Angola"[Mesh]) OR "Benin"[Mesh]) OR "Botswana"[Mesh]) OR "Burkina Faso"[Mesh]) OR "Burundi"[Mesh]) OR "Cameroon"[Mesh]) OR "Cape Verde"[Mesh]) OR "Central African Republic"[Mesh]) OR "Chad"[Mesh]) OR "Congo"[Mesh]) OR "Cote d'Ivoire"[Mesh]) OR "Democratic Republic of the Congo"[Mesh]) OR "Djibouti"[Mesh]) OR "Egypt"[Mesh]) OR "Equatorial Guinea"[Mesh]) OR "Eritrea"[Mesh]) OR "Ethiopia"[Mesh]) OR "Gabon"[Mesh]) OR "Gambia"[Mesh]) OR "Ghana"[Mesh]) OR "Guinea-Bissau"[Mesh]) OR "Guinea"[Mesh]) OR "Kenya"[Mesh]) OR "Lesotho"[Mesh]) OR "Liberia"[Mesh]) OR "Libya"[Mesh]) OR "Malawi"[Mesh]) OR "Mali"[Mesh]) OR "Mauritania"[Mesh]) OR "Morocco"[Mesh]) OR "Mozambique"[Mesh]) OR "Namibia"[Mesh]) OR "Niger"[Mesh]) OR "Nigeria"[Mesh]) OR "Rwanda"[Mesh]) OR "Senegal"[Mesh]) OR "Sierra Leone"[Mesh]) OR "Somalia"[Mesh]) OR "South Africa"[Mesh]) OR "South Sudan"[Mesh]) OR "Sudan"[Mesh]) OR "Swaziland"[Mesh]) OR "Tanzania"[Mesh]) OR "Togo"[Mesh]) OR "Tunisia"[Mesh]) OR "Uganda"[Mesh]) OR "Zambia"[Mesh]) OR "Zimbabwe"[Mesh]))) AND (((("Clinical Trials as Topic"[Mesh] OR "Double-Blind Method"[Mesh] OR "Single-Blind Method"[Mesh] OR "Research Design"[Mesh] OR "Placebos"[Mesh] OR "Random Allocation"[Mesh] OR "Randomized Controlled Trials as Topic"[Mesh] OR "Double-Blind Method"[Mesh] OR "Randomized Controlled Trial"[Publication Type] OR "Clinical Trial"[Publication Type] OR "Controlled Clinical Trial"[Publication Type] OR single blind[Title/Abstract] OR single blinded[Title/Abstract] OR single masked[Title/Abstract] OR double blind[Title/Abstract] OR double blinded[Title/Abstract] OR double masked[Title/Abstract] OR triple blind[Title/Abstract] OR triple blinded[Title/Abstract] OR triple masked[Title/Abstract] OR double-blind* OR random allocation[Title/Abstract] OR random allocations[Title/Abstract] OR random allocated[Title/Abstract] OR randomly allocated[Title/Abstract] OR clinical trial[Title/Abstract] OR clinical trials[Title/Abstract] OR placebo*[Title/Abstract]))))) AND ((((((((((((((((((((((((((((((((((((((((((((((((((((((((((((((((((((("cell phone based") OR "cell phone communication") OR "cell phone intervention") OR "cell phone interventions") OR "cell phone reminders") OR "cell phone service") OR "cell phone text message") OR "cell phone text messages") OR "cell phone text messaging") OR "health app") OR "health apps") OR "health application") OR "health applications") OR "health communication") OR ipad) OR ipads) OR "mobile application") OR "mobile applications") OR mHealth) OR "mobile health") OR "mobile health application") OR "mobile health applications") OR "mobile health care") OR "mobile healthcare") OR "mobile health intervention") OR "mobile health interventions") OR "mobile phone application") OR "mobile phone applications") OR "mobile phone call") OR "mobile phone calls") OR "mobile phone communication") OR "mobile phone communications") OR "mobile phone intervention") OR "mobile phone interventions") OR "mobile phone message") OR "mobile phone messages") OR "mobile phone messaging") OR "mobile phone reminder") OR "mobile phone reminders") OR "mobile phone short message") OR "short message service") OR "short message services") OR "short messaging service") OR "short messaging services") OR smartphone) OR smart-phone) OR sms) OR "sms intervention") OR "sms interventions") OR "sms message") OR "sms messages") OR "sms messaging") OR "sms reminder") OR "sms-reminder") OR "sms reminders") OR "sms-reminders") OR "text message") OR "text messages") OR "text messaging") OR "text message based") OR "text message intervention") OR "text message interventions") OR "text message reminder") OR "text message reminders") OR "text message-reminder") OR "text message-reminders") OR texting)) OR ((((("cell phones"[MeSH Terms]) OR "health communication"[MeSH Terms]) OR "text messaging"[MeSH Terms]) OR "Reminder System"[MeSH Terms]) OR "telemedicine"[MeSH Terms]))

**Embase**

| 1. (((singl* or doubl* or treb* or tripl*) adj (blind*3 or mask$3)) or (allocated adj2 random)).tw. or (clin* adj25 trial*).ti,ab. or (clinic: adj trial$1).tw. or (double-blind* or random*).af. or exp "clinical trial (topic)"/ or exp double blind procedure/ or exp single blind procedure/ or exp triple blind procedure/ or placebo*.tw. or exp placebo/ or exp randomization/ or Random.af. or Random*.tw. or exp "randomized controlled trial (topic)"/ or randomized.ab. or randomly allocated.tw. or randomly.ab. or trial.ab. or trial.ti. or exp "controlled clinical trial (topic)"/ or randomized controlled trial/ or "randomized controlled trial (topic)"/ or exp controlled clinical trial/ |
| --- |
| 2. africa.mp. [mp=title, abstract, heading word, drug trade name, original title, device manufacturer, drug manufacturer, device trade name, keyword, floating subheading] |
| 3. "north africa".mp. |
| 4. "Africa south of the sahara".mp. |
| 5. "central africa".mp. |
| 6. "South africa".mp. |
| 7. african.mp. |
| 8. "north african".mp. |
| 9. "Central african".mp. |
| 10. "East African".mp. |
| 11. "Southern African".mp. |
| 12. "West African".mp. |
| 13. angola.mp. |
| 14. benin.mp. |
| 15. botswana.mp. |
| 16. "burkina faso".mp. |
| 17. burundi.mp. |
| 18. cameroon.mp. |
| 19. cape verde.mp. |
| 20. "central african republic".mp. |
| 21. chad.mp. |
| 22. comoros.mp. |
| 23. congo.mp. |
| 24. "cote d'ivoire".mp. |
| 25. "democratic republic congo".mp. |
| 26. djibouti.mp. |
| 27. "equatorial guinea".mp. |
| 28. eritrea.mp. |
| 29. ethiopia.mp. |
| 30. gabon.mp. |
| 31. gambia.mp. |
| 32. ghana.mp. |
| 33. guinea.mp. |
| 34. guinea-bissau.mp. |
| 35. kenya.mp. |
| 36. lesotho.mp. |
| 37. liberia.mp. |
| 38. madagascar.mp. |
| 39. malawi.mp. |
| 40. mali.mp. |
| 41. mayotte.mp. |
| 42. mozambique.mp. |
| 43. namibia.mp. |
| 44. niger.mp. |
| 45. nigeria.mp. |
| 46. rwanda.mp. |
| 47. senegal.mp. |
| 48. "sierra leone".mp. |
| 49. somalia.mp. |
| 50. "south africa".mp. |
| 51. "south sudan".mp. |
| 52. sudan.mp. |
| 53. swaziland.mp. |
| 54. tanzania.mp. |
| 55. togo.mp. |
| 56. uganda.mp. |
| 57. zambia.mp. |
| 58. zimbabwe.mp. |
| 59. algeria.mp. |
| 60. egypt.mp. |
| 61. "libyan arab jamahiriya".mp. |
| 62. mauritania.mp. |
| 63. morocco.mp. |
| 64. tunisia.mp. |
| 65. western sahara.mp. |
| 66. 2 or 3 or 4 or 5 or 6 or 7 or 8 or 9 or 10 or 11 or 12 or 13 or 14 or 15 or 16 or 17 or 18 or 19 or 20 or 21 or 22 or 23 or 24 or 25 or 26 or 27 or 28 or 29 or 30 or 31 or 32 or 33 or 34 or 35 or 36 or 37 or 38 or 39 or 40 or 41 or 42 or 43 or 44 or 45 or 46 or 47 or 48 or 49 or 50 or 51 or 52 or 53 or 54 or 55 or 56 or 57 or 58 or 59 or 60 or 61 or 62 or 63 or 64 or 65 |
| 67. "africa south of the sahara"/ or angola/ or benin/ or botswana/ or burkina faso/ or burundi/ or cameroon/ or cape verde/ or central africa/ or central african republic/ or chad/ or comoros/ or congo/ or cote d'ivoire/ or democratic republic congo/ or djibouti/ or equatorial guinea/ or eritrea/ or ethiopia/ or gabon/ or gambia/ or ghana/ or guinea/ or guinea-bissau/ or kenya/ or lesotho/ or liberia/ or madagascar/ or malawi/ or mali/ or mayotte/ or mozambique/ or namibia/ or niger/ or nigeria/ or rwanda/ or senegal/ or sierra leone/ or somalia/ or south africa/ or south sudan/ or sudan/ or swaziland/ or tanzania/ or togo/ or uganda/ or zambia/ or zimbabwe/ |
| 68. north africa/ or algeria/ or egypt/ or libyan arab jamahiriya/ or mauritania/ or morocco/ or tunisia/ or western sahara/ |
| 69. Central Africa/ |
| 70. South Africa/ |
| 71. african/ |
| 72. north african/ |
| 73. central african/ |
| 74. east african/ |
| 75. southern african/ |
| 76. west african/ |
| 77. Africa/ |
| 78. 67 or 68 or 69 or 70 or 71 or 72 or 73 or 74 or 75 or 76 or 77 |
| 79. 66 or 78 |
| 80. "cell phone based".mp. |
| 81. "cell phone communication".mp. |
| 82. "cell phone intervention".mp. |
| 83. "cell phone interventions".mp. |
| 84. "cell phone reminders".mp. |
| 85. "cell phone service".mp. |
| 86. "cell phone text message".mp. |
| 87. "cell phone text messages".mp. |
| 88. "cell phone text messaging".mp. |
| 89. "health app".mp. |
| 90. "health apps".mp. |
| 91. "health application".mp. |
| 92. "health applications".mp. |
| 93. "health communication".mp. |
| 94. ipad.mp. |
| 95. ipads.mp. |
| 96. "mobile application".mp. |
| 97. "mobile applications".mp. |
| 98. mhealth.mp. |
| 99. "mobile health".mp. |
| 100. "mobile health application".mp. |
| 101. "mobile health applications".mp. |
| 102. "mobile health care".mp. |
| 103. "mobile healthcare".mp. |
| 104. "mobile health intervention".mp. |
| 105. "mobile health interventions".mp. |
| 106. "mobile phone message".mp. |
| 107. "mobile phone messages".mp. |
| 108. "mobile phone messaging".mp. |
| 109. "mobile phone reminder".mp. |
| 110. "mobile phone reminders".mp. |
| 111. "mobile phone short message".mp. |
| 112. "short message service".mp. |
| 113. "short message services".mp. |
| 114. "short messaging service".mp. |
| 115. "short messaging services".mp. |
| 116. smartphone.mp. |
| 117. smart-phone.mp. |
| 118. sms.mp. |
| 119. "sms intervention".mp. |
| 120. "sms interventions".mp. |
| 121. "sms message".mp. |
| 122. "sms messages".mp. |
| 123. "sms messaging".mp. |
| 124. "sms reminder".mp. |
| 125. "sms-reminder".mp. |
| 126. "sms reminders".mp. |
| 127. "sms-reminders".mp. |
| 128. "text message".mp. |
| 129. "text messages".mp. |
| 130. "text messaging".mp. |
| 131. "text message based".mp. |
| 132. "text message intervention".mp. |
| 133. "text message interventions".mp. |
| 134. "text message reminder".mp. |
| 135. "text message reminders".mp. |
| 136. "text message-reminder".mp. |
| 137. "text message-reminders".mp. |
| 138. texting.mp. |
| 139. 80 or 81 or 82 or 83 or 84 or 85 or 86 or 87 or 88 or 89 or 90 or 91 or 92 or 93 or 94 or 95 or 96 or 97 or 98 or 99 or 100 or 101 or 102 or 103 or 104 or 105 or 106 or 107 or 108 or 109 or 110 or 111 or 112 or 113 or 114 or 115 or 116 or 117 or 118 or 119 or 120 or 121 or 122 or 123 or 124 or 125 or 126 or 127 or 128 or 129 or 130 or 131 or 132 or 133 or 134 or 135 or 136 or 137 or 138 |
| 140. telemedicine/ |
| 141. mobile application/ |
| 142. mobile phone/ |
| 143. text messaging/ |
| 144. reminder system/ |
| 145. 140 or 141 or 142 or 143 or 144 |
| 146. 139 or 145 |
| 147. 1 and 79 and 146 |

**CENTRAL**

| #1 | MeSH descriptor: [Africa] explode all trees |
| --- | --- |
| #2 | MeSH descriptor: [Africa, Northern] explode all trees |
| #3 | MeSH descriptor: [Africa, Central] explode all trees |
| #4 | MeSH descriptor: [Africa, Eastern] explode all trees |
| #5 | MeSH descriptor: [Africa, Western] explode all trees |
| #6 | MeSH descriptor: [Africa, Southern] explode all trees |
| #7 | MeSH descriptor: [Africa South of the Sahara] explode all trees |
| #8 | #1 or #2 or #3 or #4 or #5 or #6 or #7 |
| #9 | (((((((((((((((((((((((((((((((((((((((((((((((((((((("Africa South of the Sahara" or "Africa, Eastern") or "Africa, Northern") or "Africa, Southern") or "Africa, Western") or "Africa") or "African") or "Algeria") or "Angola") or "Benin") or "Botswana") or "Burkina Faso") or "Burundi") or "Cameroon") or "Cape Verde") or "Central African Republic") or "Chad") or "Congo") or "Cote d'Ivoire") or "Democratic Republic of the Congo") or "Djibouti") or "Egypt") or "Equatorial Guinea") or "Eritrea") or "Ethiopia") or "Gabon") or "Gambia") or "Ghana") or "Guinea-Bissau") or "Guinea") or "Kenya") or "Lesotho") or "Liberia") or "Libya") or "Malawi") or "Mali") or "Mauritania") or "Morocco") or "Mozambique") or "Namibia") or "Niger") or "Nigeria") or "Rwanda") or "Senegal") or "Sierra Leone") or "Somalia") or "South Africa") or "South Sudan") or "Sudan") or "Swaziland") or "Tanzania") or "Togo") or "Tunisia") or "Uganda") or "Zambia") or "Zimbabwe" |
| #10 | #8 or #9 |
| #12 | MeSH descriptor: [Telemedicine] 2 tree(s) exploded |
| #13 | MeSH descriptor: [Mobile Applications] explode all trees |
| #14 | MeSH descriptor: [Cell Phone] explode all trees |
| #15 | MeSH descriptor: [Smartphone] explode all trees |
| #16 | MeSH descriptor: [Text Messaging] explode all trees |
| #17 | MeSH descriptor: [Reminder Systems] explode all trees |
| #18 | MeSH descriptor: [Health Communication] explode all trees |
| #19 | #12 or #13 or #14 or #15 or #16 or #17 or #18 |
| #20 | cell phone based |
| #21 | ((((((((((((((((((((((((((((((((((((((((((((((((((((((((((((((((("cell phone based" or "cell phone communication") or "cell phone intervention") or "cell phone interventions") or "cell phone reminders") or "cell phone service") or "cell phone text message") or "cell phone text messages") or "cell phone text messaging") or "health app") or "health apps") or "health application") or "health applications") or "health communication") or ipad) or pads) or "mobile application") or "mobile applications") or mHealth) or "mobile health") or "mobile health application") or "mobile health applications") or "mobile health care") or "mobile healthcare") or "mobile health intervention") or "mobile health interventions") or "mobile phone application") or "mobile phone applications") or "mobile phone call") or "mobile phone calls") or "mobile phone communication") or "mobile phone communications") or "mobile phone intervention") or "mobile phone interventions") or "mobile phone message") or "mobile phone messages") or "mobile phone messaging") or "mobile phone reminder") or "mobile phone reminders") or "mobile phone short message") or "short message service") or "short message services") or "short messaging service") or "short messaging services") or smartphone) or smart-phone) or sms) or "sms intervention") or "sms interventions") or "sms message") or "sms messages") or "sms messaging") or "sms reminder") or "sms-reminder") or "sms reminders") or "sms-reminders") or "text message") or "text messages") or "text messaging") or "text message based") or "text message intervention") or "text message interventions") or "text message reminder") or "text message reminders") or "text message-reminder") or "text message-reminders") or texting |
| #22 | #20 or #21 |
| #23 | #22 and #10 |

**Global health library**

| S20 | S6 AND S15 AND S19 |
| --- | --- |
| S19 | S16 OR S17 OR S18 |
| S18 | (((((((((((((((((((((((((((((((((((((((((((((((((((((((((((((((((("cell phone based") OR "cell phone communication") OR "cell phone intervention") OR "cell phone interventions") OR "cell phone reminders") OR "cell phone service") OR "cell phone text message") OR "cell phone text messages") OR "cell phone text messaging") OR "health app") OR "health apps") OR "health application") OR "health applications") OR "health communication") OR ipad) OR pads) OR "mobile application") OR "mobile applications") OR mHealth) OR "mobile health") OR "mobile health application") OR "mobile health applications") OR "mobile health care") OR "mobile healthcare") OR "mobile health intervention") OR "mobile health interventions") OR "mobile phone application") OR "mobile phone applications") OR "mobile phone call") OR "mobile phone calls") OR "mobile phone communication") OR "mobile phone communications") OR "mobile phone intervention") OR "mobile phone interventions") OR "mobile phone message") OR "mobile phone messages") OR "mobile phone messaging") OR "mobile phone reminder") OR "mobile phone reminders") OR "mobile phone short message") OR "short message service") OR "short message services") OR "short messaging service") OR "short messaging services") OR smartphone) OR smart-phone) OR sms) OR "sms intervention") OR "sms interventions") OR "sms message") OR "sms messages") OR "sms messaging") OR "sms reminder") OR "sms-reminder") OR "sms reminders") OR "sms-reminders") OR "text message") OR "text messages") OR "text messaging") OR "text message based") OR "text message intervention") OR "text message interventions") OR "text message reminder") OR "text message reminders") OR "text message-reminder") OR "text message-reminders") OR texting |
| S17 | DE "telecommunications" |
| S16 | DE "telephones" |
| S15 | S13 OR S14 |
| S14 | ((((((((((((((((((((((((((((((((((((((((((((((((((((((("Africa South of the Sahara") OR "Africa, Eastern") OR "Africa, Northern") OR "Africa, Southern") OR "Africa, Western") OR "Africa") OR “African”) OR "Algeria") OR "Angola") OR "Benin") OR "Botswana") OR "Burkina Faso") OR "Burundi") OR "Cameroon") OR "Cape Verde") OR "Central African Republic") OR "Chad") OR "Congo") OR "Cote d'Ivoire") OR "Democratic Republic of the Congo") OR "Djibouti") OR "Egypt") OR "Equatorial Guinea") OR "Eritrea") OR "Ethiopia") OR "Gabon") OR "Gambia") OR "Ghana") OR "Guinea-Bissau") OR "Guinea") OR "Kenya") OR "Lesotho") OR "Liberia") OR "Libya") OR "Malawi") OR "Mali") OR "Mauritania") OR "Morocco") OR "Mozambique") OR "Namibia") OR "Niger") OR "Nigeria") OR "Rwanda") OR "Senegal") OR "Sierra Leone") OR "Somalia") OR "South Africa") OR "South Sudan") OR "Sudan") OR "Swaziland") OR "Tanzania") OR "Togo") OR "Tunisia") OR "Uganda") OR "Zambia") OR "Zimbabwe" |
| S13 | S7 OR S8 OR S9 OR S10 OR S11 OR S12 |
| S12 | DE "Africa" |
| S11 | DE "Egypt" OR DE "Libya" OR DE "Maghreb" |
| S10 | DE "Southern Africa" OR DE "Botswana" OR DE "Comoros" OR DE "Lesotho" OR DE "Mozambique" OR DE "Namibia" OR DE "Saint Helena" OR DE "South Africa" OR DE "Swaziland" OR DE "Angola" OR DE "Zambia" OR DE "Zimbabwe" |
| S9 | DE "West Africa" OR DE "Benin" OR DE "Burkina Faso" OR DE "Cape Verde" OR DE "Cote d'Ivoire" OR DE "Gambia" OR DE "Ghana" OR DE "Guinea" OR DE "Guinea-Bissau" OR DE "Liberia" OR DE "Mali" OR DE "Mauritania" OR DE "Niger" OR DE "Nigeria" OR DE "Senegal" OR DE "Sierra Leone" OR DE "Togo" OR DE "Western Sahara" OR DE "Sahel" |
| S8 | DE "East Africa" OR DE "Djibouti" OR DE "Eritrea" OR DE "Ethiopia" OR DE "Kenya" OR DE "Madagascar" OR DE "Malawi" OR DE "Rwanda" OR DE "Seychelles" OR DE "Somalia" OR DE "Sudan" OR DE "Tanzania" OR DE "Uganda" |
| S7 | DE "Central Africa" OR DE "Africa South of Sahara" OR DE "Burundi" OR DE "Cameroon" OR DE "Central African Republic" OR DE "Chad" OR DE "Congo" OR DE "Congo Democratic Republic" OR DE "Equatorial Guinea" OR DE "Gabon" OR DE "Sao Tome and Principe" |
| S6 | S1 OR S2 OR S3 OR S4 OR S5 |
| S5 | AB "random allocation" OR AB "random allocations" OR AB "random allocated" OR AB "randomly allocated" OR AB "clinical trials" OR AB placebo OR AB placebos OR AB "drug therapy" OR "review of reported cases" OR "multicase review" OR "practice guideline" |
| S4 | "randomized controlled trial" OR "Clinical Trial" OR "Controlled Clinical Trial" OR AB "single blind" OR AB "single blinded" OR AB "single masked" OR AB "double blind" OR AB "double blinded" OR AB "double masked" OR AB "triple blind" OR "triple blinded" OR "triple masked" |
| S3 | DE "reviews" |
| S2 | DE "placebos" |
| S1 | DE "randomized controlled trials" OR DE "clinical trials" |
