# Supplementary material for: One-way SMS and healthcare outcomes in Africa: Systematic review of randomised trials with meta-analysis
Source: PLoS One. 2019 Jun 6;14(6):e0217485. doi: 10.1371/journal.pone.0217485 (PMC6553734; doi:10.1371/journal.pone.0217485)

**S2 File: Subgroup and sensitivity analyses of one-way SMS versus no SMS on healthcare appointment attendance**

Fig A: Effect of one-way SMS versus no SMS on healthcare appointment attendance: Sensitivity analysis: Fixed effect model

**
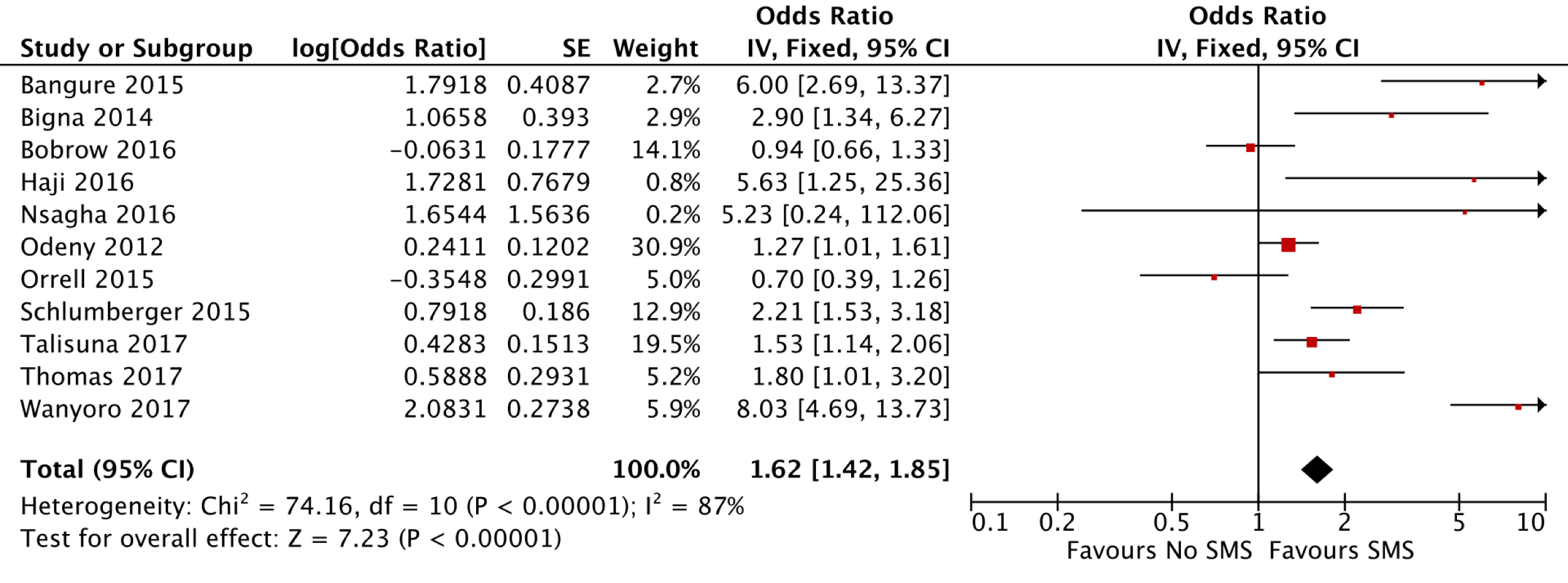
**

Fig B: Effect of one-way SMS versus no SMS on healthcare appointment attendance: Sensitivity analysis: Cluster trial excluded


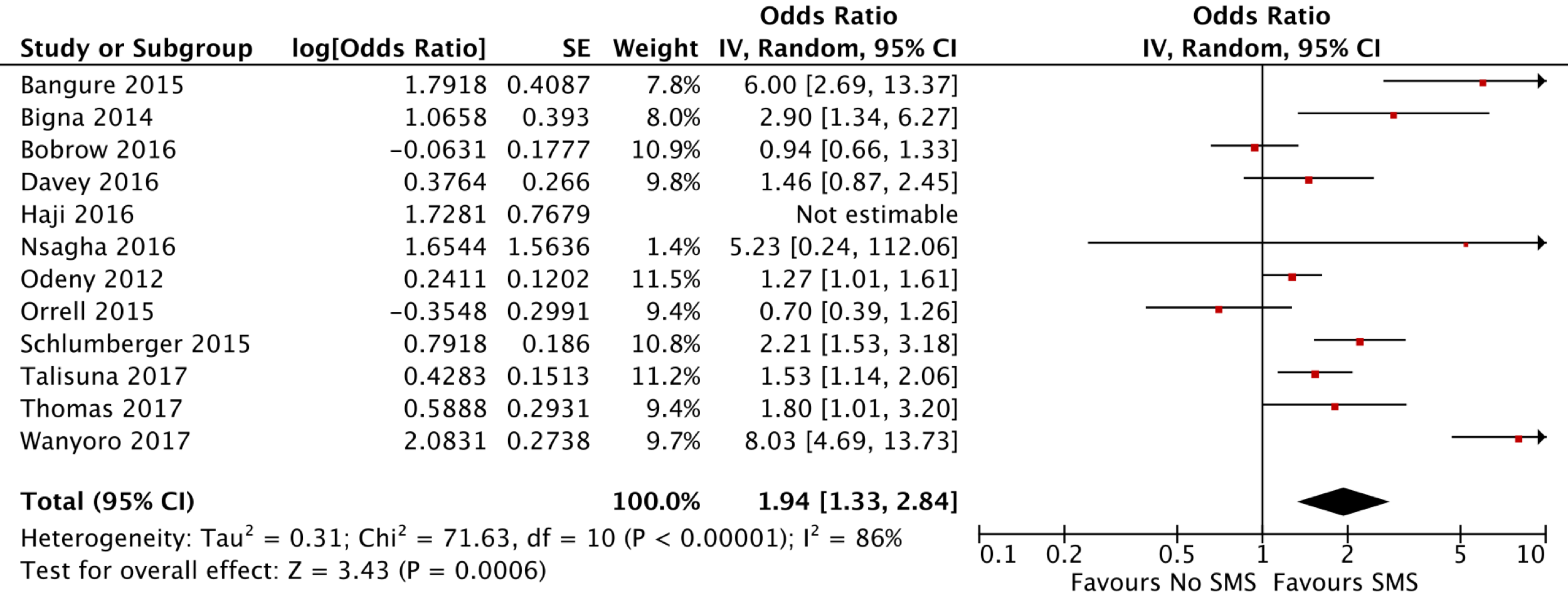


Fig C: Effect of one-way SMS versus no SMS on healthcare appointment attendance: Sensitivity analysis: Outliers excluded

Fig D: Effect of one-way SMS versus no SMS on healthcare appointment attendance: Subgroup analysis: Low risk of bias versus high risk of bias trials


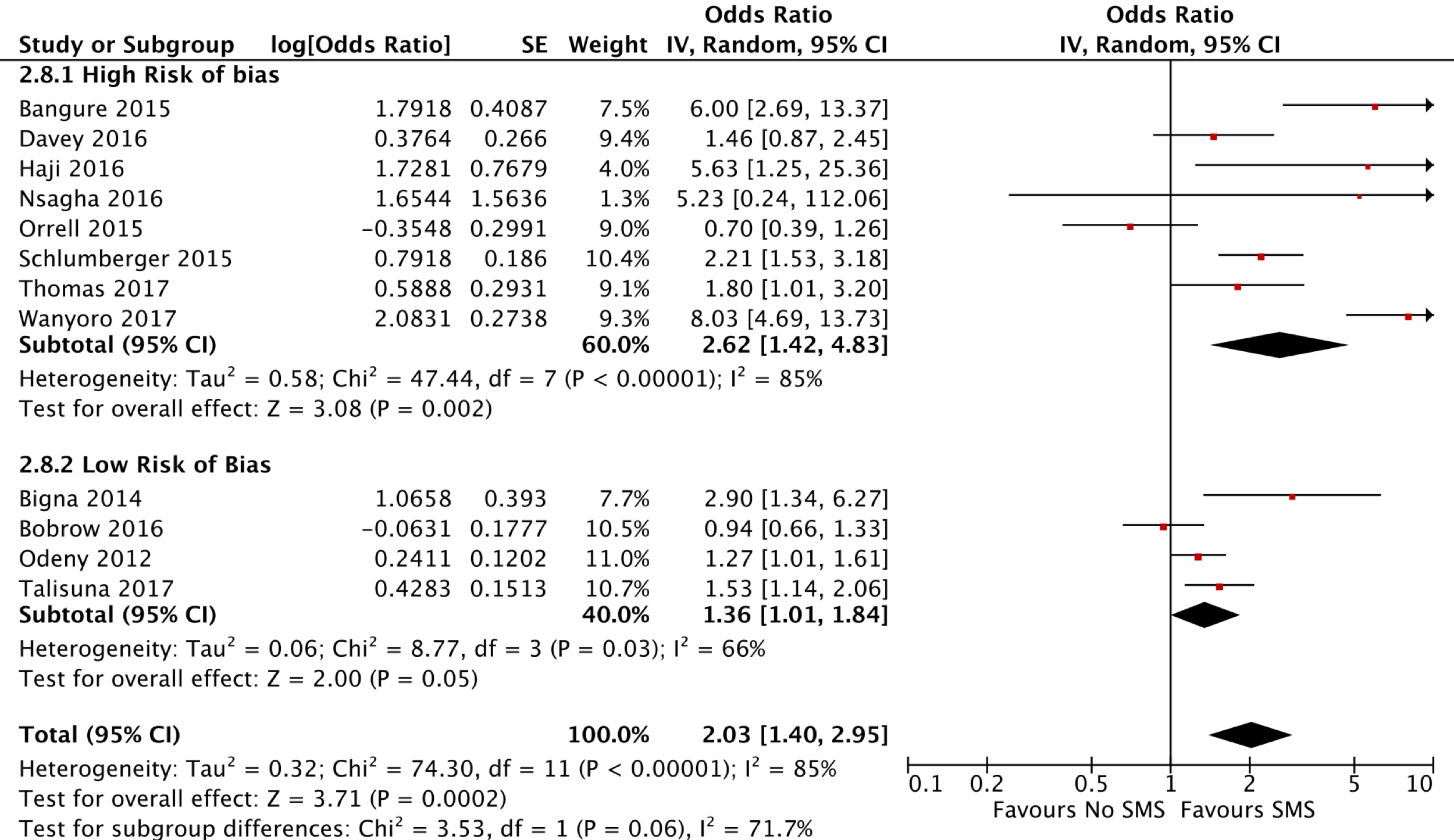


Fig E: Effect of one-way SMS versus no SMS on healthcare appointment attendance: Subgroup analysis: Clinical level


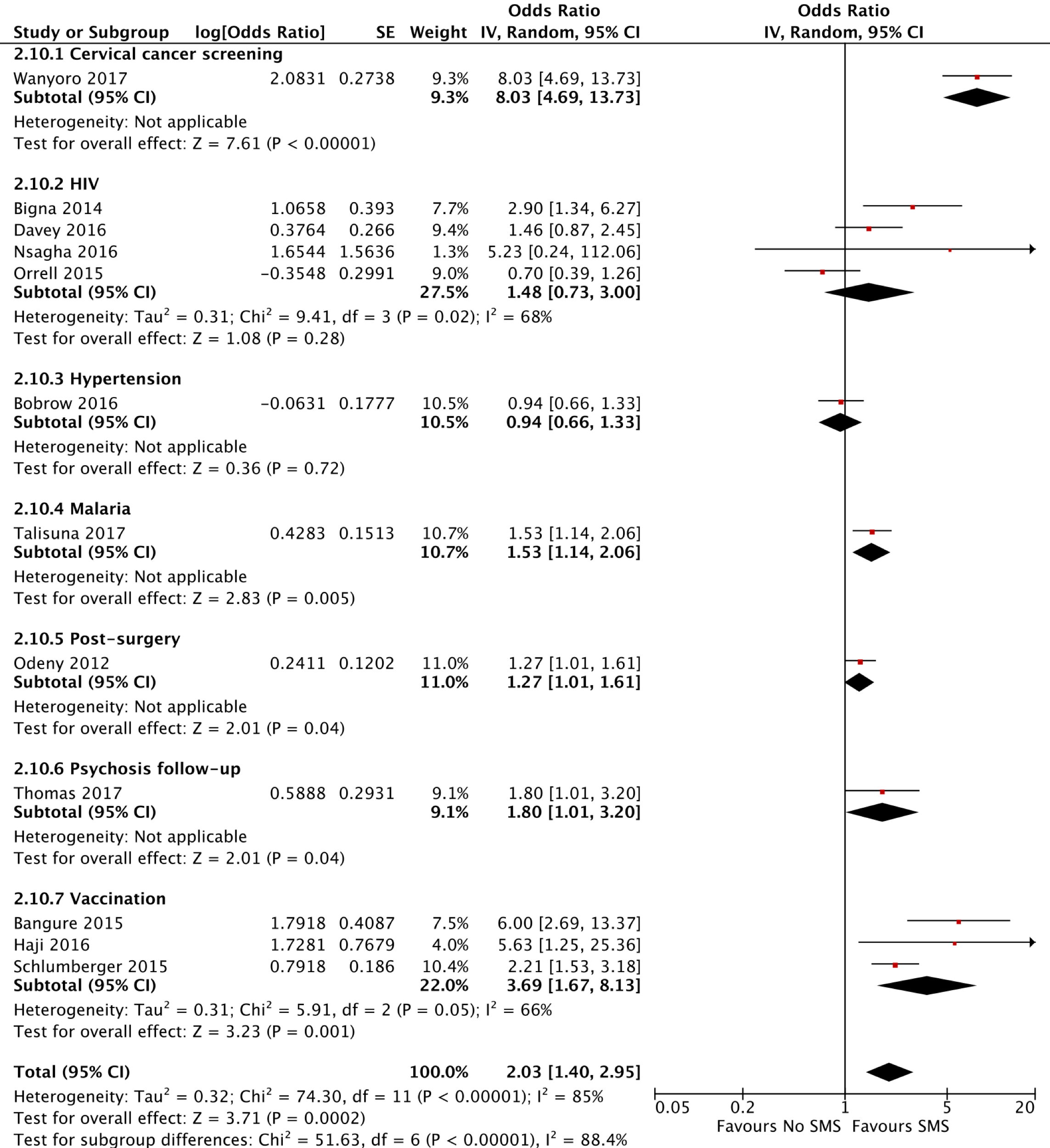

Supplement: S2 File — (DOCX) [file pone.0217485.s002.docx]
