# Supplementary material for: One-way SMS and healthcare outcomes in Africa: Systematic review of randomised trials with meta-analysis
Source: PLoS One. 2019 Jun 6;14(6):e0217485. doi: 10.1371/journal.pone.0217485 (PMC6553734; doi:10.1371/journal.pone.0217485)
Supplement: S3 File — (DOCX) [file pone.0217485.s003.docx]

**S3 File: Subgroup and sensitivity analyses of one-way SMS versus no SMS on medicine adherence**

Fig F: Effect of one-way SMS versus no SMS on medicine adherence: Sensitivity analysis: Fixed effect model

Fig G: Effect of one-way SMS versus no SMS on medicine adherence: Subgroup analysis: Low risk of bias versus high risk of bias trials

Fig H: Effect of one-way SMS versus no SMS on medicine adherence: Subgroup analysis: Clinical level


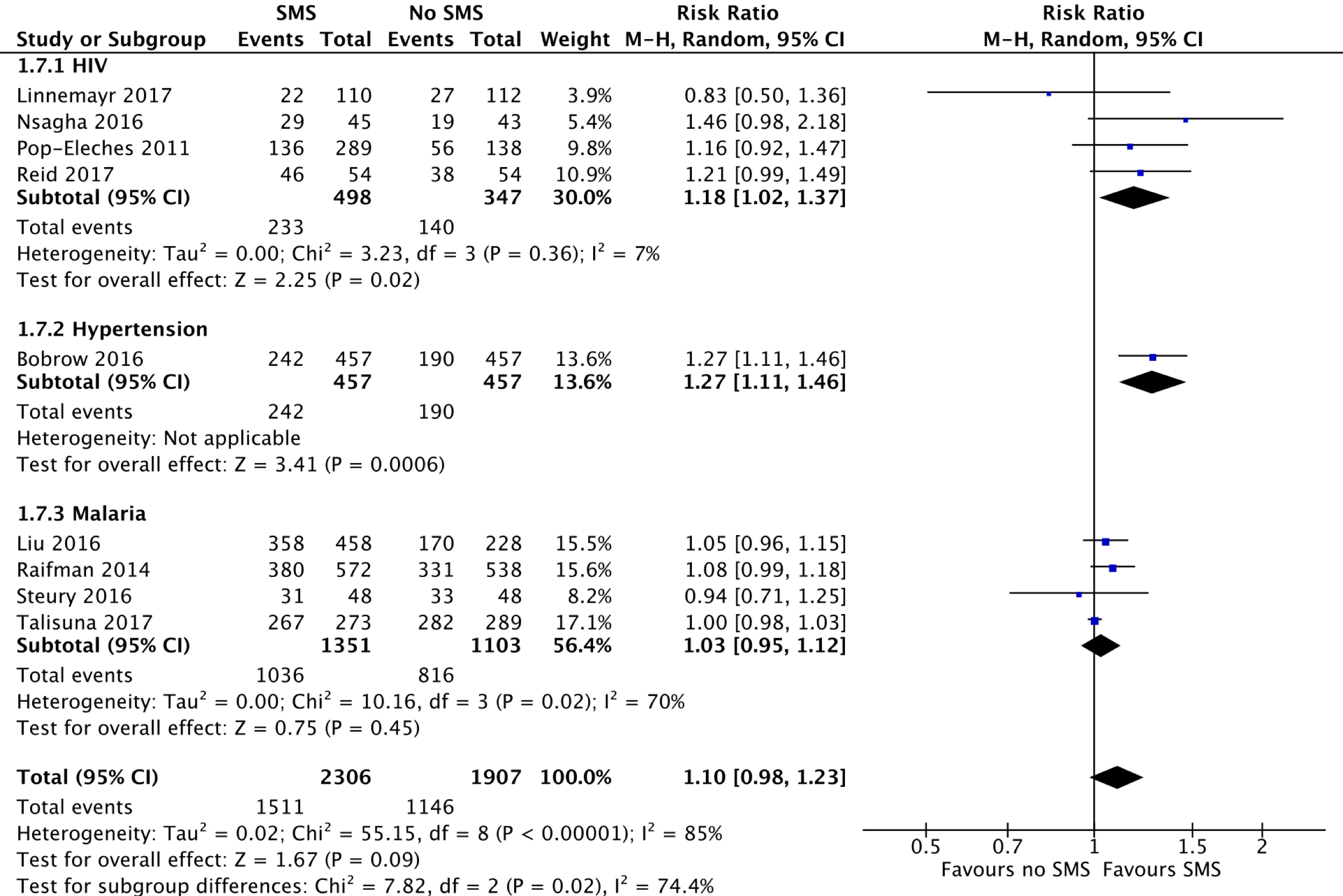


Fig I: Effect of one-way SMS versus no SMS on medicine adherence: Sensitivity analysis: Outlier excluded
