# Supplementary material for: One-way SMS and healthcare outcomes in Africa: Systematic review of randomised trials with meta-analysis
Source: PLoS One. 2019 Jun 6;14(6):e0217485. doi: 10.1371/journal.pone.0217485 (PMC6553734; doi:10.1371/journal.pone.0217485)
Supplement: S4 File — (DOCX) [file pone.0217485.s004.docx]

**S4 File: Risk of bias assessment**

### **Bangure 2015**

|  | Bias  *Outcome: Attendance* | Authors’ judgement | Support for judgement |  |
| --- | --- | --- | --- | --- |
|  |  |  |  |  |
|  | Random sequence generation (selection bias) | Low risk | Participants were assigned to study arms via computer-generated random numbers. |  |
|  |  |  |  |  |
|  | Allocation concealment (selection bias) | Unclear risk | Not stated. |  |
|  | Blinding of personnel (performance bias) | Unclear risk | Not stated. |  |
|  |  |  |  |  |
|  | Blinding of outcome assessment  (detection bias) | Unclear risk | Not stated. |  |
|  | Selective reporting (reporting bias) | High risk | Trial is registered retrospectively once follow-up has finished (ISRCTN70918594). |  |

### **Bigna 2014**

|  | Bias  *Outcome: Attendance* | Authors’ judgement | Support for judgement |  |
| --- | --- | --- | --- | --- |
|  |  |  |  |  |
|  | Random sequence generation (selection bias) | Low risk | ”Randomisation and allocation were done centrally with WinPepi version 11.25. Eligible participants (adult–child pairs) were randomly assigned in blocks of four and allocated (1:1:1:1) sequentially in the order of receipt of a randomisation code.” |  |
|  |  |  |  |  |
|  | Allocation concealment (selection bias) | Low risk | ”Randomisation and allocation were done centrally with WinPepi version 11.25. Eligible participants (adult–child pairs) were randomly assigned in blocks of four and allocated (1:1:1:1) sequentially in the order of receipt of a randomisation code.” |  |
|  | Blinding of personnel (performance bias) | Low risk | “The treating physician, the medical administrative assistant responsible for contacting participants in the intervention groups 2, 3, and 4 via mobile phone, the nurse (outcome assessor) responsible for recording the patient’s presence or absence at the appointment, and the data analysts were all masked to group assignment.” |  |
|  |  |  |  |  |
|  | Blinding of outcome assessment  (detection bias) | Low risk | The outcome assessor and data analysist were blinded to group assignment. |  |
|  | Selective reporting (reporting bias) | Low risk | Trial registered two months after enrolment started (PACTR201304000528276). The study’s specified outcomes have been reported. |  |

### **Bobrow, 2016**

|  | Bias  *Outcome: Adherence* | Authors’ judgement | Support for judgement |  |
| --- | --- | --- | --- | --- |
|  |  |  |  |  |
|  | Random sequence generation (selection bias) | Low risk | "A software algorithm assigned participants independently of the research team to information-only adherence support, interactive adherence support, or usual care in a 1:1:1 ratio using a nondeterministic minimization algorithm to ensure balance between groups with respect to age, sex, baseline SBP, years with hypertension, and recent clinic attendance." |  |
|  |  |  |  |  |
|  | Allocation concealment (selection bias) | Low risk | "Trained research staff collected baseline data immediately before enrolment, and an independent administrator entered these data into a secure, Web-based randomization database implemented by the Primary Care Clinical Trials Unit in Oxford. A software algorithm assigned participants independently of the research team" |  |
|  | Blinding of participants and personnel (performance bias) | Low risk | "Trial statisticians, researchers, clinic staff, and research assistants who collected outcome data were masked to allocated interventions until the trial database was locked." |  |
|  |  |  |  |  |
|  | Blinding of outcome assessment (detection bias) | Low risk | "Trial statisticians, researchers, clinic staff, and research assistants who collected outcome data were masked to allocated interventions until the trial database was locked." |  |
|  | Incomplete outcome data (attrition bias) | Low risk | "Attrition rates did not differ significantly between groups, and those randomized were broadly similar to those screened but not randomized, although more were black (Table III in the online-only Data Supplement)." Reasons for exclusions due to loss to follow-up is described for all arms independently. |  |
|  |  |  |  |  |
|  | Selective reporting (reporting bias) | Low risk | "The trial was registered with the South African National Clinical Trials Register (SANCTR DOH-27-1212-386) before recruitment began. This registration was subsequently incorporated within the Pan African Trial Register (PACTR201411000724141), and we registered with ClinicalTrials.gov (NCT02019823) at the time of publishing the protocol to ensure that details were widely available".  It is not possible to access the SANCTR registry and double check predefined outcomes. The PACTR registration is registered 18 months into the study after the first participants have returned for follow-up, similar to the NCT registry. The specified outcomes in the trial registry as well as the ones in the published protocol have been reported. |  |

### **Davey 2016**

|  | Bias  *Outcome: Attendance* | Authors’ judgement | Support for judgement |  |
| --- | --- | --- | --- | --- |
|  |  |  |  |  |
|  | Random sequence generation (selection bias) | Low risk | Electronic randomisation list. |  |
|  |  |  |  |  |
|  | Allocation concealment (selection bias) | Low risk | "To guarantee allocation concealment, prior to enrolment per health facility an electronic randomisation list was generated with 50% probability to belong to one arm. Only the study’s statistician was aware of the study allocation. At recruitment the counsellor invited and enrolled patients unware of the randomization allocation." |  |
|  | Blinding of personnel (performance bias) | Low risk | "All clinicians and investigators were blinded to the study allocation". |  |
|  |  |  |  |  |
|  | Blinding of outcome assessment (detection bias) | Low risk | "All study staff and clinicians were blinded to randomization" |  |
|  | Selective reporting (reporting bias) | High risk | Trial is registered retrospectively once follow-up had finished (NCT01910493). |  |

### **Haji 2016**

|  | Bias  *Outcome: Attendance* | Authors’ judgement | Support for judgement |  |
| --- | --- | --- | --- | --- |
|  |  |  |  |  |
|  | Random sequence generation (selection bias) | Unclear risk | Cluster randomisation using “simple-random sampling”. Not stated how randomisation is undertaken. |  |
|  |  |  |  |  |
|  | Allocation concealment (selection bias) | Unclear risk | Not stated. |  |
|  | Blinding of personnel (performance bias) | Unclear risk | Not stated. |  |
|  |  |  |  |  |
|  | Blinding of outcome assessment (detection bias) | Unclear risk | Not stated. |  |
|  | Selective reporting (reporting bias) | Unclear risk | No protocol or registry information available. |  |
|  | Baseline imbalance | High risk | Low number of clusters: One cluster per arm in three health districts. |  |
|  | Recruitment bias | High risk | Recruitment occurred after randomisation of clusters |  |

### **Linnemayr, 2017**

|  | Bias  *Outcome: Adherence* | Authors’ judgement | Support for judgement |  |
| --- | --- | --- | --- | --- |
|  |  |  |  |  |
|  | Random sequence generation (selection bias) | Low risk | "Participants were randomly assigned by simple randomization in a 1-to-1-to-1 ratio to control group (care as usual) or 1 of 2 SMS intervention groups: 1-way SMS (message only) or 2-way SMS (message plus response option) via a random number generator based on lists of eligible clients in the electronic records." |  |
|  |  |  |  |  |
|  | Allocation concealment (selection bias) | High risk | "The principal investigator (S. L.) conducted the randomization." |  |
|  | Blinding of participants and personnel (performance bias) | Low risk | "Study participants and the study coordinators could not be masked to intervention allocation because participants had to be informed of their study group assignment, and on the basis of that, either received text messages or did not."  During routine home visits "study coordinators did not provide feedback to participants regarding their MEMS measured adherence to minimize potential confounding of intervention effects with “MEMS-use” effects." |  |
|  |  |  |  |  |
|  | Blinding of outcome assessment (detection bias) | Low risk | The outcome was assessed via an electronic medication event monitoring system (MEMS) cap - electronic pillbox. The investigators performing the data analyses were masked to study group assignment. |  |
|  | Incomplete outcome data (attrition bias) | Low risk | "The analysis of primary outcomes was by intention to treat. [...] we classified all individuals lost to follow-up as nonadherent for the period since their last recorded MEMS opening. Our secondary analysis was per-protocol (complete-case) analysis of outcomes, in which we included only participants who had complete MEMS outcome data."  "In the 48 weeks following enrolment, 17 people left the study (loss to follow-up, transferred to a different clinic, or died) and 28 did not have MEMS outcomes collected throughout because of broken or malfunctioning MEMS, losing their MEMS caps, or repeatedly not bringing it to clinic visits. These constitute 13.6% of participants who were enrolled." |  |
|  |  |  |  |  |
|  | Selective reporting (reporting bias) | High risk | Trial registered 20 months after trial started, and after the follow-up period has finished for some participants (NCT00830622). The following specified outcomes were not reported: RNA-suppression, Retention in care, Quality of life, Health. |  |

### **Liu, 2016**

|  | Bias  *Outcome: Adherence* | Authors’ judgement | Support for judgement |  |
| --- | --- | --- | --- | --- |
|  |  |  |  |  |
|  | Random sequence generation (selection bias) | Unclear risk | "To ensure that none of the survey staff would know which participants were chosen to receive the SMS, the study manager, who did not have any interaction with participants, randomly assigned surveys into the treatment groups after the surveys were returned to the study office each day. The protocol for treatment assignment entailed assigning consecutive surveys to one of the intervention or control arms on the day of the baseline survey, replicating the randomization procedure from the previous study (Modrek et al. 2014).  According to the Modrek et al. 2014 trial, "the protocol for treatment assignment entailed assigning every other survey to the SMS treatment group on the day of the baseline survey". However, in the Modrek trial, there were only two arms and not three arms as in this trial. Therefore, it is unclear how the sequence generation actual occurred in this trial. |  |
|  |  |  |  |  |
|  | Allocation concealment (selection bias) | Low risk | The allocation occurred on the study office each day after study participants had been enrolled. |  |
|  | Blinding of participants and personnel (performance bias) | Low risk | The survey staff did not know the randomisation status, however the study manager did. |  |
|  |  |  |  |  |
|  | Blinding of outcome assessment (detection bias) | Low risk | They survey staff called study participants four days after the baseline survey to obtain information on whether or not they had taken the medicine. |  |
|  | Incomplete outcome data (attrition bias) | Low risk | "Thirteen participants were excluded from the analysis either because they did not provide a phone number (n=11) or refused to allow a RDT to be administered (n=2). The remaining 728 participants were randomized to one of three arms. A total of 686 (94.2%) participants were followed-up for the phone survey. The reasons for loss to follow-up is not stated, however, the number excluded in each arm is described. As the number is low it is not expected to influence the outcome. |  |
|  |  |  |  |  |
|  | Selective reporting (reporting bias) | High risk | The trial is registered prospectively (ISRCTN12605216). The specified outcomes in trial registry are:  Primary outcome measure: Malaria rapid diagnostic test result measured at baseline  Secondary outcome measures, assessed 4 days after enrolment and testing will include: 1. Self-reported treatment with anti-malarial medication 2. Self-reported treatment with non-antimalarial medications 3. Perceptions of drug shop vendors ability to perform rapid diagnostic tests 4. Perceptions of rapid diagnostic tests in general 5. Cost effectiveness 6. Perceptions of the text message reminders.  The outcomes stated in the article are:  1. Followed treatment advice 2. Took anti-malarial drug 3. Took symptom drug  4. Increased agreement that chemists or pharmacists ‘should be allowed to do RDTs |  |

### **Nsagha 2016**

|  | Bias  *Outcome: Attendance and adherence* | Authors’ judgement | Support for judgement |  |
| --- | --- | --- | --- | --- |
|  |  |  |  |  |
|  | Random sequence generation (selection bias) | Low risk | “All the 90 participants were recruited before they were randomly allocated to the two groups. Using this serially numbered list of 90 participants, ballots were prepared and we randomly drew out numbers without replacing until we got 45 patients in group A and 45 in group B.” |  |
|  |  |  |  |  |
|  | Allocation concealment (selection bias) | High risk | The researchers have not explicitly ensured themselves against the risk of drawing a new number or excluding a patient with "wrong" number. |  |
|  | Blinding of personnel (performance bias) | Unclear risk | Not stated. |  |
|  |  |  |  |  |
|  | Blinding of outcome assessment (detection bias) | High risk | The outcome is based on self-reports in a questionnaire. |  |
|  | Incomplete outcome data (attrition bias) | Low risk | Two patients from the control group could not provide information on treatment duration and adherence to medicine. |  |
|  | Selective reporting (reporting bias) | Unclear risk | No protocol or registry information available. |  |

### **Pop-Eleches, 2011**

|  | Bias  *Outcome: Adherence* | Authors’ judgement | Support for judgement |  |
| --- | --- | --- | --- | --- |
|  |  |  |  |  |
|  | Random sequence generation (selection bias) | Low risk | "The randomization schedule was prepared in advance of enrolment by the investigators. A sequence of random numbers between 0 and 1 were generated, and four equal intervals between 0 and 2/3 corresponded to the four intervention groups, whereas the value interval from 2/3 to 1 corresponded to the control group." |  |
|  |  |  |  |  |
|  | Allocation concealment (selection bias) | Low risk | "One-third of the sample was allocated to the control group, and the remaining two-thirds of the sample were allocated evenly to each of the four intervention groups." |  |
|  | Blinding of participants and personnel (performance bias) | Unclear risk | Not stated. |  |
|  |  |  |  |  |
|  | Blinding of outcome assessment (detection bias) | Low risk | It is not stated if the outcome assessor is blinded. However, as the outcome was assessed via an electronic pillbox monitor, it is unlikely that it would influence the outcome. |  |
|  | Incomplete outcome data (attrition bias) | Low risk | "Participants in the study were defined as lost to follow-up if more than 90 days had elapsed after their last recorded MEMS [ed. pillbox] opening. Sixty-nine participants, or 16% of the original sample, were lost to follow-up as of 31 December 2008. This is consistent -with typical loss to follow-up rates in similar programs [27,28]. The percentage of participants lost to follow- up by group -was as follows: control group (14.4%), short daily reminders group (18.6%), long daily reminders group (16.7%), short weekly reminders group (22%), and long -weekly reminders group (10.8%). There was no significant difference in the rate of loss to follow-up across the control and each of the four intervention groups (P=0.48)."  "Our primary analysis was performed on an intention-to-treat basis. Individuals who discontinued therapy or who were lost to follow-up were classified as nonadherent. [...] Our secondary analysis was per-protocol [...] [and] participants lost to follow-up during a given period were not included in the analysis for that period." |  |
|  |  |  |  |  |
|  | Selective reporting (reporting bias) | High risk | No published protocol and trial registered retrospectively once follow-up had finished (NCT01058694). The study’s specified outcomes have been reported. |  |

### **Odeny 2012**

|  | Bias  *Outcome: Attendance* | Authors’ judgement | Support for judgement |  |
| --- | --- | --- | --- | --- |
|  |  |  |  |  |
|  | Random sequence generation (selection bias) | Low risk | ”A block randomization scheme with variable blocks of size 4–16 was generated using Stata ralloc.ado module v3.5.2. Randomization was stratified by clinic.” |  |
|  |  |  |  |  |
|  | Allocation concealment (selection bias) | Low risk | “A biostatistician in Seattle who was not involved in any other aspect of the study implementation developed the randomisation scheme.”  “Investigators and study staff were blinded to the block number, block size, and sequence in the block. Individual participant randomization envelopes were shipped from Seattle to Kisumu, while the key to intervention assignments was retained in Seattle. Participants were assigned to intervention arms using pre- prepared sequentially numbered, sealed, opaque envelopes containing group assignment. Study staff issued the next envelope in the series.” |  |
|  | Blinding of personnel (performance bias) | Low risk | Investigators and study staff were blinded to the block number, block size, and sequence in the block. |  |
|  |  |  |  |  |
|  | Blinding of outcome assessment (detection bias) | Low risk | The clinicians and nurses performing the circumcision procedure and follow-up were blinded to study group assignment. |  |
|  | Selective reporting (reporting bias) | Low risk | Trial registered prospectively (NCT01186575). The study’s specified outcomes have been reported. |  |

### **Orrell, 2015**

|  | Bias  *Outcome: Attendance* | Authors’ judgement | Support for judgement |  |
| --- | --- | --- | --- | --- |
|  |  |  |  |  |
|  | Random sequence generation (selection bias) | Low risk | "The random number sequence and envelopes were generated off-site." |  |
|  |  |  |  |  |
|  | Allocation concealment (selection bias) | Low risk | "Allocation to study arm was concealed in sealed individual opaque envelopes, which were numbered from 1 to 230 and opened consecutively after a participant met study entry criteria. [...] The envelopes were opened by the study nurse, blinded to the allocation, onsite." |  |
|  | Blinding of participants and personnel (performance bias) | High risk | "Staff (both study and clinic) and participants were not masked to arm allocation after randomization." |  |
|  |  |  |  |  |
|  | Blinding of outcome assessment (detection bias) | High risk | Retention in care is measured through attendance to clinical appointments.  "Staff (both study and clinic) and participants were not masked to arm allocation after randomization."  "A tracking list was generated for all missed visits at the end of each month. Individuals were added to this list if they were more than 4 weeks late for an appointment [...]. All those on the community tracking list were called by a community care worker and, if they could not be contacted by phone, visited at home. This process was repeated every week for up to 3 attempts." |  |
|  | Selective reporting (reporting bias) | High risk | The trial was registered 14 months after enrolment started (PACTR201311000641402) - after some patients had returned for follow-up. The study’s specified outcomes have been reported. |  |

### **Raifman, 2014**

|  | Bias  *Outcome: Adherence* | Authors’ judgement | Support for judgement |  |
| --- | --- | --- | --- | --- |
|  |  |  |  |  |
|  | Random sequence generation (selection bias) | High risk | "Participants were sequentially (in order of their enrolment) assigned to treatment and control, with every other enrolled subject assigned to either treatment or control in an alternating manner. The text messaging system then further randomized participants within the treatment condition to receive message A or message A+B with equal probability, based on a pseudo-random number draw." |  |
|  |  |  |  |  |
|  | Allocation concealment (selection bias) | Unclear risk | Not stated. |  |
|  | Blinding of participants and personnel (performance bias) | Low risk | "Enumerators interacting with subjects during the baseline and follow-up interviews were not aware of treatment assignment" |  |
|  |  |  |  |  |
|  | Blinding of outcome assessment (detection bias) | High risk | "The primary outcome variable in this study was self-reported completion of the ACT treatment regimen, with the interviewee reporting whether the patient completed all of their doses." |  |
|  | Incomplete outcome data (attrition bias) | Low risk | Four participants (0.4%) were lost to follow-up, and 26 (2.3%) were excluded due to missing data on treatment completion. Data was analysed as intention-to-treat. |  |
|  |  |  |  |  |
|  | Selective reporting (reporting bias) | High risk | Trial was registered 11 months into the trial, two months before follow-up finished (NCT01722734). The study’s specified outcome has been reported. |  |

### **Reid, 2017**

|  | Bias  *Outcome: Adherence* | Authors’ judgement | Support for judgement |  |
| --- | --- | --- | --- | --- |
|  |  |  |  |  |
|  | Random sequence generation (selection bias) | Unclear risk | Not stated. |  |
|  |  |  |  |  |
|  | Allocation concealment (selection bias) | Low risk | "Randomization, laboratory assays, and analyses were done by investigators masked to treatment allocation" |  |
|  | Blinding of participants and personnel (performance bias) | Low risk | "Randomization, laboratory assays, and analyses were done by investigators masked to treatment allocation" |  |
|  |  |  |  |  |
|  | Blinding of outcome assessment (detection bias) | Low risk | "Randomization, laboratory assays, and analyses were done by investigators masked to treatment allocation" |  |
|  | Incomplete outcome data (attrition bias) | Low risk | A total of 108 participants were enrolled, randomized and included in all subsequent analysis. There was no loss to follow-up among these participants. An additional 20 participated but were not randomised and excluded from the study. |  |
|  |  |  |  |  |
|  | Selective reporting (reporting bias) | High risk | Trial was registered retrospectively once follow-up had finished (NCT01001741). Only the primary outcome is specified in the trial registry, while the secondary outcomes are not stated. |  |

### **Schlumberger 2015**

|  | Bias  *Outcome: Attendance* | Authors’ judgement | Support for judgement |  |
| --- | --- | --- | --- | --- |
|  |  |  |  |  |
|  | Random sequence generation (selection bias) | High risk | It appears that the study may be pseudo-randomised. Participants should pick a number and if they picked an odd number, they would be assigned to the intervention arm. |  |
|  |  |  |  |  |
|  | Allocation concealment (selection bias) | Unclear risk | Not stated. |  |
|  | Blinding of personnel (performance bias) | Unclear risk | Not stated. |  |
|  |  |  |  |  |
|  | Blinding of outcome assessment (detection bias) | Unclear risk | Not stated. |  |
|  | Selective reporting (reporting bias) | Unclear risk | No protocol or registry information available. |  |

### **Steury, 2016**

|  | Bias  *Outcome: Adherence* | Authors’ judgement | Support for judgement |  |
| --- | --- | --- | --- | --- |
|  |  |  |  |  |
|  | Random sequence generation (selection bias) | Unclear risk | "Each participant was randomly assigned to either the intervention group or the control group by block randomization through an envelope system."  It is further stated that a research assistant "initiated the SMS intervention sequence", but it is not stated in what way the sequence generation occurred through the envelope system. |  |
|  |  |  |  |  |
|  | Allocation concealment (selection bias) | Unclear risk | Not stated apart from use of an "envelope system". |  |
|  | Blinding of participants and personnel (performance bias) | High risk | SMS' were sent directly to the participant by the primary investigator. It is not stated if the research assistant, who enrolled the participants, was blinded. |  |
|  |  |  |  |  |
|  | Blinding of outcome assessment (detection bias) | Low risk | The outcome assessor is the primary investigator, who was not blinded. However, outcome is assessed via the Medication Event Monitoring System (MEMS) - an electronic pill box - which is an objective measurement tool. |  |
|  | Incomplete outcome data (attrition bias) | Low risk | A total of 96 participants were enrolled and outcome data is available for all participants. However, eight participants (four from control group and four from intervention group) did not return the electronic pill box. |  |
|  |  |  |  |  |
|  | Selective reporting (reporting bias) | Unclear risk | No protocol or registry information available. |  |

### **Talisuna 2017**

|  | Bias  *Outcome: Attendance and adherence* | Authors’ judgement | Support for judgement |  |
| --- | --- | --- | --- | --- |
|  |  |  |  |  |
|  | Random sequence generation (selection bias) | Low risk | "The randomization codes were generated by an offsite statistician and randomization numbers were applied in sequence of recruitment".  Additional pseudo-randomisation within intervention arms to allocate to timing of home visits. |  |
|  |  |  |  |  |
|  | Allocation concealment (selection bias) | Low risk | The randomization codes were generated by offsite |  |
|  | Blinding of personnel (performance bias) | Low risk | "Participants and nurses performing home visits could not be masked. The study personnel at health facilities were necessarily aware of categories for assigning day of home visits but were blind to the intervention arm." |  |
|  |  |  |  |  |
|  | Blinding of outcome assessment (detection bias) | Low risk | Adherence was assessed by the nurses performing the home visits by use of pill counts and caregivers' reports. |  |
|  | Incomplete outcome data (attrition bias) | Low risk | "PP analysis after excluding 62 patients who reported not receiving SMS in the intervention group and 14 protocol violations showed very similar results to the ITT analysis" |  |
|  | Selective reporting (reporting bias) | Low risk | Trial registered prospectively (ISRCTN39512726). The study’s specified outcomes have been reported. |  |

### **Thomas 2017**

|  | Bias  *Outcome: Attendance* | Authors’ judgement | Support for judgement |  |
| --- | --- | --- | --- | --- |
|  |  |  |  |  |
|  | Random sequence generation (selection bias) | Low risk | Randomisation codes were generated by use of computer software (Research Randomizer). |  |
|  |  |  |  |  |
|  | Allocation concealment (selection bias) | Low risk | Allocation occurred by a researcher who was blind to the recruitment. |  |
|  | Blinding of personnel (performance bias) | Unclear risk | Blinding of participants were not possible due to the overt nature of the intervention. Not stated if personnel were blinded. |  |
|  |  |  |  |  |
|  | Blinding of outcome assessment (detection bias) | High risk | The first author is the outcome assessor and checks patient files for attendance. However, he is also the one responsible for sending the SMS. |  |
|  | Selective reporting (reporting bias) | High risk | Protocol available (PACTR201511001358312), retrospectively registered. The study’s specified outcomes have been reported. |  |

### **Wanyoro 2017**

|  | Bias  *Outcome: Attendance* | Authors’ judgement | Support for judgement |  |
| --- | --- | --- | --- | --- |
|  |  |  |  |  |
|  | Random sequence generation (selection bias) | Low risk | "Randomization was conducted using a computer-based randomization program [...]. Using the participant’s mobile phone numbers, a 1:1 computer generated random allocation was performed allocating participants into an intervention arm (4 SMS reminders) and control arm (no SMS reminders). [...] A software expert developed and administered the system and was not blinded to the study." |  |
|  |  |  |  |  |
|  | Allocation concealment (selection bias) | Low risk | The "allocation was concealed and blinded to the principal investigator and his assistant". |  |
|  | Blinding of personnel (performance bias) | Low risk | The "allocation was concealed and blinded to the principal investigator and his assistant". |  |
|  |  |  |  |  |
|  | Blinding of outcome assessment (detection bias) | Unclear risk | Not stated. |  |
|  | Selective reporting (reporting bias) | High risk | The trial was registered prospectively (PACTR201305000526384). Trial registration is noted as "not recruiting yet", however corresponding author has sent publication from finished trial.  The secondary outcome of "Women with low grade lesion who return for re-screening at 6 months" is not reported. Further, the trial registration state two identical primary and secondary outcomes: "[...]completion of a repeat Pap test at 12 months from baseline" and "Women who comply with recommendation of repeat Pap test after 12 months".  The primary outcome shows a dramatic effect and the article is published in a journal by a publisher on Beall’s list of predatory journals and publishers. |  |
