# Supplementary material for: One-way SMS and healthcare outcomes in Africa: Systematic review of randomised trials with meta-analysis
Source: PLoS One. 2019 Jun 6;14(6):e0217485. doi: 10.1371/journal.pone.0217485 (PMC6553734; doi:10.1371/journal.pone.0217485)
Supplement: S5 File — (PDF) [file pone.0217485.s005.pdf]

PROSPERO
International prospective register of systematic reviews

National Institute for Health Research

Landscaping the field of randomised controlled trials of one-way SMS interventions in Africa and analysis of interventions targeted clients' attendance to health care appointments and medicine adherence: a systematic review

Ditte Søndergaard Linde, Malene Korsholm, Johnson Katanga, Vibeke Rasch, Andreas Lundh, Marianne Skovsager Andersen

Citation

Ditte Søndergaard Linde, Malene Korsholm, Johnson Katanga, Vibeke Rasch, Andreas Lundh, Marianne Skovsager Andersen. Landscaping the field of randomised controlled trials of one-way SMS interventions in Africa and analysis of interventions targeted clients' attendance to health care appointments and medicine adherence: a systematic review. PROSPERO 2018 CRD42018081062 Available from:
http://www.crd.york.ac.uk/PROSPERO/display_record.php?ID=CRD42018081062

Review question

The aim of this review is to landscape the number of randomised controlled trials in Africa involving SMS interventions where the intervention is compared to standard care. Further, an analysis is conducted of how effective SMS interventions are at improving attendance to health appointments and medicine adherence are compared to standard care.

Problem/Population: Africa

Intervention: SMS

Comparison: Standard Care

Outcome: Attendance to health care appointments / Adherence to medicine.

Searches

The primary author developed the specific search strategies in co-operation with an experienced clinical research librarian. Firstly, an electronic search for headings and sub-headings was conducted so that the search string would include non-indexed studies. Literature searches will be updated before final analysis to retrieve recently published studies.
PubMed: 27 March 2017
Global Health: 28 March 2017

Cochrane: 27 March 2017

Embase: 27 March 2017

Updated literature search was conducted on all databases at 28 April 2018.

Selected literature reviews be manually searched for additional literature. We will search reference lists of included trials for additional trials. Further, UN and World Bank reports manually searched for studies concerning mobile health. We will search www.ClinicalTrials.gov, the WHO trial registry ICTRP, and PACTR for ongoing and unpublished trials.

Search strategy

https://www.crd.york.ac.uk/PROSPEROFILES/81062_STRATEGY_20181127.pdf

Types of study to be included

We will include randomized controlled trials (RCTs), in any language, including cluster RCTs and pilot

studies. All other study-design will be excluded.

Condition or domain being studied

The effect of one-way SMS interventions on health appointment attendance and medicine adherence in Africa.

Participants/population

Inclusion criteria:

- Male and female healthcare clients/patients.

- Guardians for healthcare clients, e.g. parents for child patients.

Exclusion criteria:

-Health personnel and healthcare providers.

Intervention(s), exposure(s)

Inclusion criteria :

• Designed to assess the effect of SMS interventions on participants healthcare behaviour, such as appointment attendance or medicine adherence.

• At least one SMS intervention arm that is exclusive SMS, such as SMS reminders, health educative SMS or SMS quiz

• A control group which receives standard care.

If co-interventions (e.g. written materials supplied to participants) are received by participants in both intervention and control arms we consider this to be part of standard care, and such studies will be included.If we find any studies that are described as pilot studies, these we will be included if they live up o the other inclusion criteria.

Exclusion criteria:

• SMS intervention arm is interactive. For example, if a study has two arms (1) SMS and (2) standard care, but the SMS service is interactive, meaning the participant can respond to the SMS and receive e.g. a phone call from a health professional, this study will be excluded.

• SMS intervention arm is integrated with other services, such as phone calls or face-to-face education. For example, a study that has a two-arm intervention with (1) SMS + phone call versus (2) standard care, this study will be excluded. However, if a study has a three-arm intervention with (1) SMS, (2) phone call and (3) standard care, this study will be included as it is possible to evaluate the isolated effect of the SMS.

Comparator(s)/control

A control group which receives standard care.

Context

The included randomised controlled trials must be conducted in Africa. Multicentre studies will be included if separate data is available.

Main outcome(s)

We will include any randomised trial meeting the inclusion criteria above irrespective of which outcomes are reported in the identified publication.

Primary outcome (outcome measure for data analysis) :

- Healthcare appointment attendance

- Medicine adherence

For outcomes reported as composite measures, we aim to extract individual outcomes when possible.

Additional outcome(s)

Secondary outcome (outcome measures for landscaping) :

- Change in health behaviour as defined by trial authors, for example healthcare appointment attendance, treatment adherence, clinical outcomes

Data extraction (selection and coding)

The reference management software Endnote X8.0.2 (Thomson Reuters, New York, NY, USA) will be used to organise articles identified in the search. Search results from difference database will be combined in an Endnote library and duplicate records will be removed. The remaining literature will be uploaded to Covidence which is a systematic review tool designed to facilitate the process of screening and enable two reviewers to work efficiently through the steps of a systematic review. Covidence will be used to manage the second duplicate search, title-abstract screening and full text screening.

Using Covidence, two reviewers (DSL, JK) will independently screen titles and abstracts of all retrieved articles for inclusion. Any inconsistency in the identification of potentially relevant papers will be discussed until consensus can be reached. For studies selected for full text screening, two reviewers (DLS, MK) will independently assess the full text articles for inclusion. Disagreements will be resolved through discussion and if consensus cannot be reached and arbiter (AL) will make the final decision.

One author (DSL) extract data and a second author (MK) verifies data extraction for trial outcomes. Data will be extracted into Excel. The extraction will include:

- Name of first author

- Name of journal and publication year

- Study objective

- Design (e.g. individual or cluster randomised trial)

- Inclusion/exclusion criteria

- Study period

- Sample size

- Description of experimental intervention

- Description of control intervention

- Length of follow-up

- Study outcomes and event data

- Trial registration

Risk of bias (quality) assessment

Two reviewers (DSL, MK) will independently will independently assess trials for risk of bias using the Cochrane Risk of Bias Tool. We will assess contamination bias and assess the following domains: random sequence generation, allocation concealment, blinding of participants and personnel, blinding of outcome assessment, incomplete outcome data, selective reporting and other biases. For cluster randomised trials we

will assess risk of bias related to recruitment bias and baseline imbalance. Disagreements will be resolved through discussion and if consensus cannot be reached and arbiter (AL) will make the final decision.

Strategy for data synthesis

We will analyse all data by performing intention-to-treat analysis using available case analysis. Using Review Manager 5 (RevMan 2012), we will calculate pooled RRs and estimate 95% confidence intervals (CIs) using the random-effects model with the Mantel-Haenszel method for dichotomous data. We will use random-effects model instead of a fixed effect model due to the anticipated clinical heterogeneity of trials.

For cluster randomized trials we will include data in meta-analysis, if possible, using methods described in Cochrane Handbook on calculating effective sample sizes.

We assessed statistical heterogeneity by using I^2.

Analysis of subgroups or subsets

We will conduct a subgroup analysis to compare low risk of bias trials with high risk of bias trials. We defined low risk of bias trials as trials with low risk of selection bias, detection bias and selective reporting, and all other trials as having high risk of bias.

Further, we will conduct a subgroup analysis on level of disease. We will conduct a subgroup analysis to compare trials randomized on individual level with trials using cluster randomization.

To test the robustness of our findings, we will perform a sensitivity analysis in which we re-analyse our primary outcome using a fixed-effect instead of a random-effects model. Further, we will conduct sensitivity analyses (1) where we exclude cluster trials, and (2) on clinical level.

Contact details for further information

Ditte Søndergaard Linde
dsondergaard@health.sdu.dk

Organisational affiliation of the review

University of Southern Denmark
www.sdu.dk

Review team members and their organisational affiliations

Mrs Ditte Søndergaard Linde. Odense University Hospital and University of Southern Denmark
Mrs Malene Korsholm. Odense University Hospital and University of Southern Denmark
Mr Johnson Katanga. Ocean Road Cancer Institute
Professor Vibeke Rasch. Odense University Hospital and University of Southern Denmark
Mr Andreas Lundh. Odense University Hospital and Hvidovre Hospital
Professor Marianne Skovsager Andersen. Odense University Hospital and University of Southern Denmark

Type and method of review

Intervention, Systematic review

Anticipated or actual start date

27 March 2017

Anticipated completion date

30 November 2018

Funding sources/sponsors

All authors were financed through their institutions: Institute of Clinical Research, University of Southern Denmark (SDU); Center for Evidence-Based Medicine, Odense University Hospital (OUH); Department of Medical Endocrinology, OUH; Department for Cancer Prevention Services, Ocean Road Cancer Institute. The funders have not had any role in developing the protocol.

Conflicts of interest

AL, MK and JK have no conflicts of interest. DSL, VR, MSA are currently conducting an RCT in Tanzania

assessing the effect of SMS interventions on attendance to cervical cancer screening follow-up appointment attendance. As of December 2017 the enrolment has finished and follow-up is on-going.
Yes

Language
English

Country
Denmark

Stage of review
Review Ongoing

Subject index terms status
Subject indexing assigned by CRD

Subject index terms
Africa; Appointments and Schedules; Delivery of Health Care; Humans

Date of registration in PROSPERO
10 January 2018

Date of publication of this version
27 November 2018

Details of any existing review of the same topic by the same authors

Stage of review at time of this submission

Stage	Started	Completed
Preliminary searches	Yes	Yes
Piloting of the study selection process	Yes	Yes
Formal screening of search results against eligibility criteria	Yes	Yes
Data extraction	Yes	Yes
Risk of bias (quality) assessment	Yes	Yes
Data analysis	Yes	No

Versions
10 January 2018
19 June 2018
29 October 2018
27 November 2018

PROSPERO
This information has been provided by the named contact for this review. CRD has accepted this information in good faith and registered the review in PROSPERO. The registrant confirms that the information supplied for this submission is accurate and complete. CRD bears no responsibility or liability for the content of this registration record, any associated files or external websites.
